# Coexisting picoplankton experience different relative grazing pressures across an ocean productivity gradient

**DOI:** 10.1073/pnas.2220771120

**Published:** 2023-10-23

**Authors:** Michael R. Landry, Michael R. Stukel, Karen E. Selph, Ralf Goericke

**Affiliations:** ^a^Scripps Institution of Oceanography, University of California at San Diego, San Diego, CA 92093; ^b^Earth, Ocean, and Atmospheric Science Department, Florida State University, Tallahassee, FL 32306; ^c^Department of Oceanography, University of Hawai’i at Manoa, Honolulu, HI 96822

**Keywords:** growth rate, grazing mortality, shared predation, heterotrophic bacteria, *Synechococcus*

## Abstract

Picophytoplankton populations [*Prochlorococcus*, *Synechococcus* (SYN), and picoeukaryotes] are dominant primary producers in the open ocean and projected to become more important with climate change. Their fates can vary, however, with microbial food web complexities. In the California Current Ecosystem, picophytoplankton biomass and abundance peak in waters of intermediate productivity and decrease at higher production. Using experimental data from eight cruises crossing the pronounced CCE trophic gradient, we tested the hypothesis that these declines are driven by intensified grazing on heterotrophic bacteria (HBAC) passed to similarly sized picophytoplankton via shared predators. Results confirm previously observed distributions as well as significant increases in bacterial abundance, cell growth, and grazing mortality with primary production. Mortalities of picophytoplankton, however, diverge from the bacterial mortality trend such that relative grazing rates on SYN compared to HBAC decline by 12-fold between low and high productivity waters. The large shifts in mortality rate ratios for coexisting populations are not explained by size variability but rather suggest high selectivity of grazer assemblages or tightly coupled tradeoffs in microbial growth advantages and grazing vulnerabilities. These findings challenge the long-held view that protistan grazing mainly determines overall biomass of microbial communities while viruses uniquely regulate diversity by “killing the winners”.

## Significance

Mortality interactions are less understood than growth processes in marine microbial food webs but equally important for determining population increases or decreases with changing environmental conditions. Experimental studies in the California Current reveal new insights about complex microbial trophic interactions across ocean productivity gradients. High production is shown to drive intensified grazing on heterotrophic bacteria, but shared predation does not transfer up-shifted mortality to co-occurring picophytoplankton of similar size. We found order-of-magnitude variability in mortality ratios indicative of highly selective predators or environmental selection for microbes with tightly coupled tradeoffs in growth advantages and grazing vulnerabilities. Study results challenge simplistic representations of mortality processes in marine ecosystem models and clear distinctions between virus and grazer roles in diversity maintenance.

Picophytoplankters, comprising the photosynthetic bacteria *Prochlorococcus* (PRO) and *Synechococcus* (SYN) as well as <2-µm phototrophic eukaryotes (PEUK), are collectively the smallest and most abundant of the ocean’s primary producers and account for most productivity over vast open-ocean areas with low nutrient concentrations (1, 2). Picophytoplankton importance is generally expected to increase with ocean warming and stratification associated with climate change. Nonetheless, Earth system and niche partitioning models diverge on whether this will increase or decrease total phytoplankton biomass and production in various regions (3–5), with neither model type accounting for the interactive complexities of microbial food webs. In the California Current, for example, high abundances of PEUK are predicted in coastal upwelling areas by a neural network–based niche model utilizing the habitat variables of temperature, light, and nutrients (5). In contrast, field data from the same region suggest that high protistan grazing pressure depresses abundances of picophytoplankton populations in the upwelling areas (6, 7).

The southern California Current Ecosystem (CCE) spans variability from open-ocean oligotrophy to upwelling eutrophy over distances that research ships can travel in a day or less and can consequently serve as a model system for studying trophic interactions that apply to a wide spectrum of ocean productivity conditions. Across the CCE gradient, biomasses of all picophytoplankton groups peak at intermediate levels of trophic richness and decline in richer waters (6–8). The mechanism advanced to explain these declines, the Enhanced Microbial Loop (EML) hypothesis (7), postulates that growth and activity of heterotrophic bacteria (HBAC) increase strongly in richer areas due to high levels of dissolved organic carbon (DOC) produced by dominant large autotrophs, which stimulates grazing on HBAC and indirectly increases grazing mortality on similarly sized picophytoplankton.

The EML hypothesis is consistent with the originally defined microbial loop function of HBAC in utilizing and returning DOC to the food web (9) as well as the strong relationships observed between HBAC and primary production (PP) in aquatic systems globally (10, 11). In the CCE, the hypothesis is also supported by fourfold to fivefold increases in both HBAC and phagotrophic flagellates, the presumptive major grazers of pico-sized cells (12, 13), which parallel the rise of large (>20-µm) phytoplankton to >75% of total autotrophic biomass and the decline of picophytoplankton to <1% (7). Nonetheless, the mechanistic inferences derived from these standing stock distributions have not been tested and confirmed by actual rate measurements. Especially critical to the EML hypothesis is shared predation, a concept that strongly links the mortalities of similarly sized HBAC and picophytoplankton to the grazing activities of their common predators (14–16).

Here, we test the EML hypothesis using rate measurements of growth and grazing for picophytoplankton and HBAC across the California Current trophic gradient from eight process cruises of the CCE-LTER (Long Term Ecological Research) Program. Using measured rates of PP as the trophic richness metric, we validate many elements of the hypothesis but find that it breaks down in translating the elevated rates of grazing mortality on HBAC to picophytoplankton via shared predation. To the contrary, intensified grazing in richer waters falls selectively on HBAC, effectively punishing the growth rate winners and sustaining diversity. Thus protistan grazers exhibit roles generally ascribed to viruses (17–19).

## Results and Discussion

### Environmental Relationships.

Environmental sampling and rate measurements were conducted on eight 3 to 4-wk process cruises of the CCE-LTER Program from May 2006 to June 2017 (Table 1). Cruises P0605, P0704, and P0810 studied stock and rate relationships in distinct homogeneous water masses (20, 21). Cruises P1106 and P1208 investigated mesoscale variability across ocean fronts (22). Cruises P1408 and P1604 sampled the system during anomalous warm-water oligotrophic conditions associated with the 2014 heat wave (23, 24) and the 2015-16 El Niño. Cruise P1706 was designed to follow the rapid offshore transport of a coastal bloom in an upwelling filament (25). Individual cruises span greater than order-of-magnitude variabilities in nutrients, Chl*a*, PP and abundances of picoplankton populations (Table 1). Collectively, the 103 experiments capture much of the system’s seasonal, spatial, and interannual variability of microbial growth and grazing mortality over the decade of study.

**Table 1.** Ranges of environmental variables and cell abundances of microbial populations measured on eight CCE process cruises in the southern CCE

Cruise	Mon/y	Exp (#)	Temp (°C)	Nitrate (µM)	Chl*a* (mg m^−3^)	Prim Prod (mg C m^−3^ d^−1^)	Abundance (10^3^ cells ml^−1^)			
							HBAC	PRO	SYN	PEUK
P0605	May 2006	18	11.3–16.4	0.04–13.5	0.10–6.5	4.5–367	710–2,480	0–191	3.5–29	2.6–27
P0704	April 2007	11	11.5–14.3	0.06–11.1	0.18–3.6	9.0–263	740–1,040	0–95	4.5–16	6.8–37
P0810	October 2010	16	14.7–17.6	0.06–2.6	0.19–1.8	6.2–84	374–3,310	0–323	4.2–92	6.0–45
P1106	June 2011	12	13.6–16.1	0.00–8.2	0.11–2.7	4.4–49	311–2,260	0–136	2.9–111	7.9–40
P1208	August 2012	10	14.3–16.8	0.01–2.5	0.09–5.8	2.8–72	373–6,140	6.7–191	2.2–246	2.6–32
P1408	August 2014	15	15.3–19.2	0.01–0.4	0.10–1.9	1.8–15	701–2,300	5.3–165	1.7–110	0.8–16
P1604	April 2016	9	12.8–16.0	0.03–6.7	0.11–7.6	2.2–115	271–4,400	0–255	7.3–113	2.3–145
P1706	June 2017	12	12.0–14.9	0.99–12.1	0.31–13.3	27.4–299	912–3,030	0–0	1.3–11	5.3–50

Cruises are noted by identification number and the main month and year of sampling. Exp (#) is the number of experimental measurement profiles conducted on each cruise. For each measured parameter, the range (presented as min-max) are the lowest (min) and highest (max) mean values for the upper euphotic zone for all profiles done on that cruise.

**Fig. 1.**
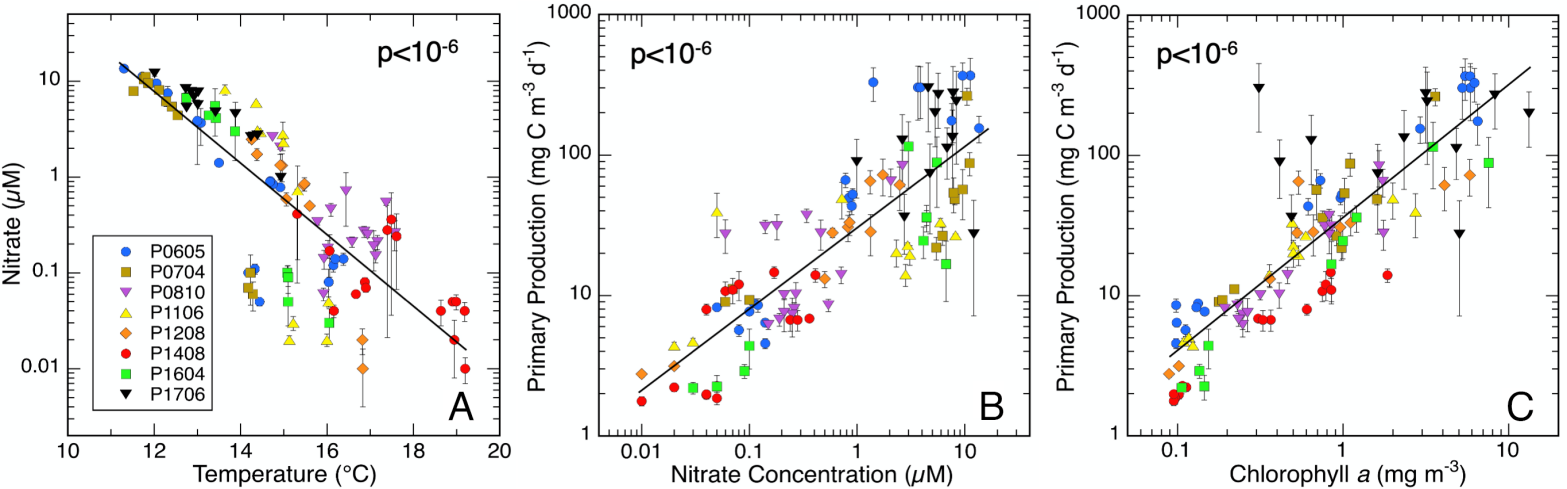
Relationships between nitrate concentration and temperature (*A*), primary production and nitrate (*B*) and primary production and chlorophyll *a* (*C*) during CCE process cruises in the southern CCE. Data are the averages of 3 to 4 depths of sampling in the upper euphotic zone (upper 26 m on average) at each experimental location; uncertainties are SEM values. Regression slopes with *P* values ≤ 0.05 are significant. Full regression statistics are in *SI Appendix*, Table S1.

Measured cruise variables show environmental relationships consistent with a wind-driven upwelling ecosystem (26). New nutrients (nitrate) enter the upper euphotic zone by upward transport or mixing of cooler deeper water along the coast or at ocean fronts and are therefore negatively related to temperature (Fig. 1*A*). PP responds strongly to nitrate concentration (Fig. 1*B*), as does Chl*a* biomass (not shown). PP and Chl*a* are consequently well correlated to one another (Fig. 1*C*), validating the use of Chl*a* as a proxy for system productivity, as was assumed in the original description of the EML hypothesis (7). Hereafter, we use measured PP as the preferred metric for testing hypothesized relationships of population abundances, growth, and grazing mortality rates over the range of CCE trophic conditions.

Although environmental conditions overlap broadly among cruises (Table 1), Fig. 1 also highlights some differences at the extreme ends of the spectrum. Cruises P1408 and P1604, conducted during anomalous warm years, are the main, but not exclusive, contributors to low-nutrient and low-PP data. In contrast, cruises that actively sought out the richest habitats (P0605 and P1706) provide the most data for cooler, high-nutrient and high-productivity conditions.

### Population Abundances and Growth Rates.

**Fig. 2.**
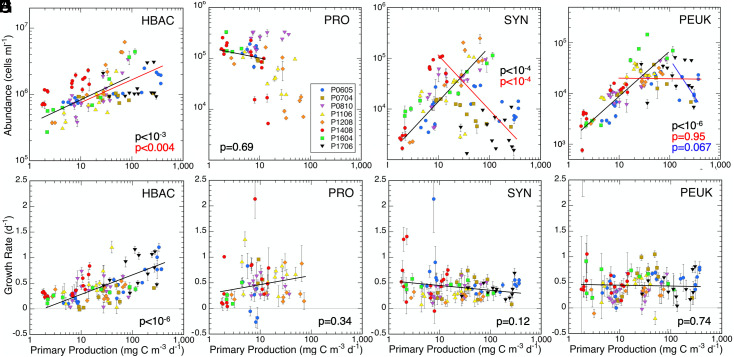
Relationships of picoplankton population abundances (*A–D*) and cell growth rates to measured PP (*E–H*) for CCE Process cruises. HBAC = heterotrophic bacteria; PRO *= Prochlorococcus*; SYN = *Synechococcus*; PEUK = photosynthetic picoeukaryotes. Cruise abbreviations correspond to Table 1. Data are mean ± SEM estimates for the upper euphotic zone. For multiple panel regressions, *P* values are color-coded to the lines plotted: PP < 100 (black), PP > 10 (red), and PP > 100 (blue). Full regression statistics are in *SI Appendix*, Table S1.

Prior observations (7) have demonstrated that picoplankton populations differ significantly in their biomass distributions across the CCE trophic gradient, with HBAC increasing uniformly as more phytoplankton biomass is added and picophytoplankton peaking at intermediate biomass values and declining at higher levels. We confirm these findings for our more limited cell abundance data by breaking them into overlapping segments of low-to-medium and medium-to-high PP (<100 and >10 mg C m^−3^ d^−1^, respectively) (Fig. 2 *A*–*D*), where intermediate PP (10 to 100 mg C m^−3^ d^−1^) corresponds to the range of Chl*a* values (~0.2 to 2 mg m^−3^) where peak picophytoplankton biomass is observed to occur (7). HBAC abundances increase significantly with PP for both PP < 100 and PP >10 mg C m^−3^ d^−1^ segments (Fig. 2*A*), consistent with the monotonic increase with trophic state reported previously (7). PRO abundances show no significant trend at very low PP (<10 mg C m^−3^ d^−1^) but drop off dramatically at higher PP, including many zero abundances not shown on the log-scale plot (Fig. 2*B* and *SI Appendix*, Table S2). SYN abundances increase significantly at the lower end of the PP scale (<100 mg C m^−3^ d^−1^) and decrease significantly at the higher end of the scale (PP > 10 mg C m^−3^ d^−1^) (Fig. 2*C*). PEUK abundances increase for PP < 100 mg C m^−3^ d^−1^and stabilize for PP > 10 mg C m^−3^ d^−1^ (Fig. 2*D*). While the PEUK data suggest an eventual abundance decline with increasing PP, we found the decrease to be marginally insignificant (too few observations) in further regression testing for very high PP > 100 mg C m^−3^ d^−1^ (Fig. 2*C*). The above results are consistent with both the trends and the previously observed order of picophytoplankton decline with increasing trophic state: PRO declines first, followed by SYN, with PEUK last and least (7).

Measured growth rates show a significant increase across the CCE trophic gradient for HBAC only (Fig. 2*E* and Table 2). Quantitatively, mean (±SEM) cell growth rates of HBAC increase 3.4 fold from 0.23 ± 0.02 d^−1^ at very low PP < 10 mg C m^−3^ d^−1^ to 0.79 ± 0.07 d^−1^ at PP > 100 mg C m^−3^ d^−1^. Growth rates for SYN and PEUK are more stable, averaging 0.48 ± 0.07 and 0.42 ± 0.08 d^−1^, respectively, for PP < 10 mg C m^−3^ d^−1^ and 0.38 ± 0.04 and 0.45 ± 0.06 d^−1^, respectively, for PP > 100 mg C m^−3^ d^−1^ (Fig. 2 *G* and *H* and Table 2). SYN growth suggests a declining trend across the PP spectrum, but the slopes are not significantly different from zero for either SYN or PEUK. PRO growth averages 0.47 ± 0.05 d^−1^ for all experiments where they were present (Fig. 2*F*). Overall, HBAC grow slower than picophytoplankton at low PP but substantially faster than picophytoplankton at high PP.

**Table 2.** Abundances and instantaneous rates of growth, grazing mortality, and net growth for HBAC, PRO, SYN, and photosynthetic picoeukaryotes (PEUK) for experiments conducted at CCE stations with low, intermediate, and high values of PP

Production/Population	Abundance (10^3^ cells mL^−1^)	Growth rate (d^−1^)	Grazing rate (d^−1^)	Net growth (d^−1^)	
PP < 10 mg C m^−3^ d^−1^					
HBAC	798 ± 65	0.23 ± 0.02	0.21 ± 0.02	0.02 ± 0.02	
PRO	153 ± 13	0.46 ± 0.08	0.38 ± 0.06	0.08 ± 0.04	
SYN	17 ± 4	0.48 ± 0.07	0.57 ± 0.09	−0.09 ± 0.05	
PEUK	6.3 ± 0.6	0.42 ± 0.08	0.46 ± 0.08	−0.04 ± 0.03	
10 < PP < 100 mg C m^−3^ d^−1^					
HBAC	1,480 ± 160	0.42 ± 0.03	0.34 ± 0.02	0.08 ± 0.02	
PRO	54 ± 13	0.48 ± 0.05	0.34 ± 0.04	0.13 ± 0.04	
SYN	49 ± 7	0.38 ± 0.02	0.30 ± 0.02	0.08 ± 0.02	
PEUK	26 ± 3	0.44 ± 0.04	0.34 ± 0.02	0.09 ± 0.04	
PP > 100 mg C m^−3^ d^−1^					
HBAC	1,820 ± 240	0.79 ± 0.07	0.61 ± 0.07	0.18 ± 0.03	
PRO	0 ± 0	nd	nd	nd	
SYN	13 ± 6	0.38 ± 0.04	0.19 ± 0.03	0.19 ± 0.03	
PEUK	22 ± 5	0.45 ± 0.06	0.30 ± 0.04	0.15 ± 0.06	

Uncertainties are SEM values.

Temperature relationships presented as *SI Appendix*, Fig. S1 provide some insights to help explain the observed differences in population abundance and growth rate trends. PRO cells, for example, are virtually absent at seawater temperatures <14 °C (*SI Appendix*, Fig. S1*A*). Thus, while shared grazing might contribute to the disappearance of PRO in productive waters, the sharpness of this decline is better explained as a low temperature effect (27, 28), below which PRO cells are physiologically unable to sustain populations in competition with other actively growing microbes. For HBAC, growth rates show negative trends with temperature for all cruises, significant for four individual cruises and for all cruises combined (*SI Appendix*, Fig. S1*B*). HBAC growth therefore appears to be held substantially below physiological potential in warmer oligotrophic waters where organic resources are limiting but more closely approaches temperature-dependent potential in colder waters with higher PP and organic cycling. In effect, the growth stimulatory effect of organic substrates for HBAC overwhelms the expected general decline of growth potential with cooling (29). In contrast, by virtue of small size and large surface area:volume ratios, picophytoplankton enjoy competitive advantages for nutrient uptake over larger primary producers, which allows them to operate closer to their physiological growth potentials on low nutrient concentrations in warm oligotrophic waters (30, 31). While such cells might still benefit from higher nutrients in productive waters, the reduced growth potential at colder temperatures substantially diminishes the effect. Thus, whether there are net growth rate advantages for SYN and PEUK in cold nutrient-rich waters likely varies with circumstances, leaving insignificant temperature trends overall for our mixed group of cruises (*SI Appendix*, Fig. S1 *C* and *D*).

### Grazing Mortality Rates and Ratios.

**Fig. 3.**
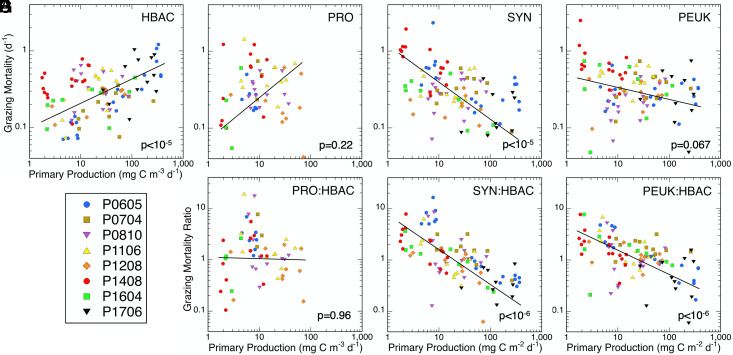
Relationships of picoplankton grazing mortality (*A–D*) and mortality ratios (*E–G*) to measured PP in the southern CCE. Cruises and populations as defined in Fig. 2 and Table 1. Regression slopes with *P* values ≤ 0.05 are significant. Full regression statistics are in *SI Appendix*, Table S1.

Grazing mortality of HBAC increases significantly with increasing PP (Fig. 3*A* and Table 2), but not for picophytoplankton populations. To the contrary, grazing mortalities trend downward at higher PP for both SYN and PEUK (Fig. 3 *C* and *D* and Table 2), most strongly and significantly for SYN. As a consequence of the different rate trends, the mortality ratios of picophytoplankton relative to HBAC decrease sharply with PP. SYN:HBAC mortality is a factor of 12 lower (0.32 ± 0.05 vs. 3.9 ± 0.6) at PP > 100 compared to PP < 10 mg C m^−3^ d^−1^ (Fig. 3*F*). PEUK:HBAC mortality ratio varies 4.2 fold (0.62 ± 0.11 vs. 2.4 ± 0.4) (Fig. 3*G*). While the regression for PRO:HBAC mortality ratios suggests a 1:1 relationship, on average, the slope is strongly influenced by very low PRO grazing rates at low PP values during the 2014 heat wave and 2016 El Niño events (Fig. 3*E*). However, the data above 4 mg C m^−3^ d^−1^ show a significant negative slope (*P* = 0.007), similar to mortality ratios for SYN and PEUK, which is a better predictor of the relative mortality trend for PRO at increasing PP. Experimental results therefore support some elements of the EML hypothesis, notably that high PP stimulates large increases in abundance, cell growth rate, and grazing turnover of HBAC. However, the hypothesis breaks down in translating the intensified mortality environment for HBAC to other microbes via rate coupling by shared predation.

The magnitude of the relative grazing shift with trophic state can best be appreciated by considering what the mortalities of SYN and PEUK would be at high PP if scaled to the mean mortality rate of HBAC in high PP waters (0.61 ± 0.07 d^−1^) and the mean mortality ratios of SYN:HBAC and PEUK:HBAC at low PP. For SYN, the scaled mortality (3.9 × 0.61 d^−1^) would be 2.38 d^−1^, which is 6.3-fold greater and net 2.0 d^−1^ higher than the measured SYN growth rate at high PP. Such a growth-mortality disparity would drive a daily 86% decline of SYN population abundance. For PEUK, mortality scaling to HBAC at high PP would be 1.46 d^−1^ or a population decline rate of 69% d^−1^. At such rates, SYN and PEUK would be driven to nominal local extinction of <1 cell mL^−1^ in 4.6 and 9.1 d, respectively.

The fact that SYN and PEUK populations persist despite the greatly intensified grazing environment for HBAC highlights the unexpected importance of shifting mortality impact in maintaining a stable niche for picophytoplankton in high PP waters. This grazing phenomenon needs to be better understood, including its variability. Comparing SYN: HBAC mortalities among different cruises (Fig. 3*F*), for example, the ratios during anomalous heat wave conditions (P1408 and P1604) fall substantially below those (i.e., higher grazing on HBAC) for normal conditions (P0605 and P1704). Such differences require more detailed consideration of circulation, biogeochemistry, and expanded food-web context but may provide insights into altered microbial dynamics and interactions associated with a warmer future ocean.

For all experiments and environmental conditions examined, the percentages of HBAC, SYN, and PEUK production consumed by micrograzers (i.e., the mean ratios of grazing mortality to growth rate) were 99 ± 15%, 100 ± 3%, and 87 ± 10%, respectively, indicating a system-level balance for picoplankton cell growth and grazing processes in the CCE overall (balance for PRO is indeterminate due to low presence at many sampling locations). Nonetheless, significant net growth of picoplankton (0.15 to 0.19 d^−1^) is evident in high productivity waters (Table 2), consistent with narrow upwelling zones close to the CCE coast being centers of biomass accumulation. This net production is transported by wind stress, mesoscale eddies, and filaments to offshore waters where it is eventually utilized (32, 33).

Because the decline of PRO in CCE coastal waters has similarities to PRO distributions in the North Pacific Transition Zone that can be explained by a shared predation model (22), we examine further here whether our experiments where PRO was present at intermediate values of 10 < PP < 100 mg C m^−3^ d^−1^ can provide any direct support for that model. This dataset comprises 27 experiments with elevated HBAC abundances and grazing mortalities (1.62 ± 0.26 × 10^6^ cells mL^−1^ and 0.39 ± 0.03 d^−1^, respectively) and where net growth rates of PRO and SYN can be compared both from the results of bottle incubations and from directly measured net changes of populations in the ambient environment from day-to-day sampling following the satellite-tracked drifter, a unique feature of our Lagrangian experimental design. Mortality rates do not differ significantly between PRO and SYN for these experiments (0.34 ± 0.04 vs. 0.31 ± 0.03 d^−1^, respectively; *P* = 0.56), but net growth rates of PRO are significantly higher than for SYN (0.13 ± 0.04 vs. 0.002 ± 0.03 d^−1^; *P* = 0.018). Observed net growth rates in the ambient mixed layer support the bottle experiment results, with neither PRO nor SYN (0.03 ± 0.10 vs. 0.04 ± 0.08 d^−1^; *P* > 0.60) showing cell abundance declines in the direction of current flow. Further, net growth rates of PRO do not decrease with increasing HBAC abundance (*P* = 0.75; *SI Appendix*, Fig. S2), and they are higher at lower PRO abundances (*P* = 0.048) suggesting a resistance to decline. Overall, where PRO were present at intermediate values of PP, they exhibited mean steady-state dynamics and provided no evidence of being driven to local extinction by elevated grazing on HBAC.

### Potential Explanations for Varying Relative Mortality Impacts.

We consider three general hypotheses to explain how relative grazing mortality impacts might vary so markedly among coexisting microbial populations with presumptive shared consumers: 1) systematic variability in relative cell sizes across the productivity gradient; 2) behavioral or compositional variability of the grazer assemblages that alter prey selectivity; and 3) environmental selection of microbial prey populations with different tradeoffs in growth rate maximization vs. predation defenses.

**Fig. 4.**
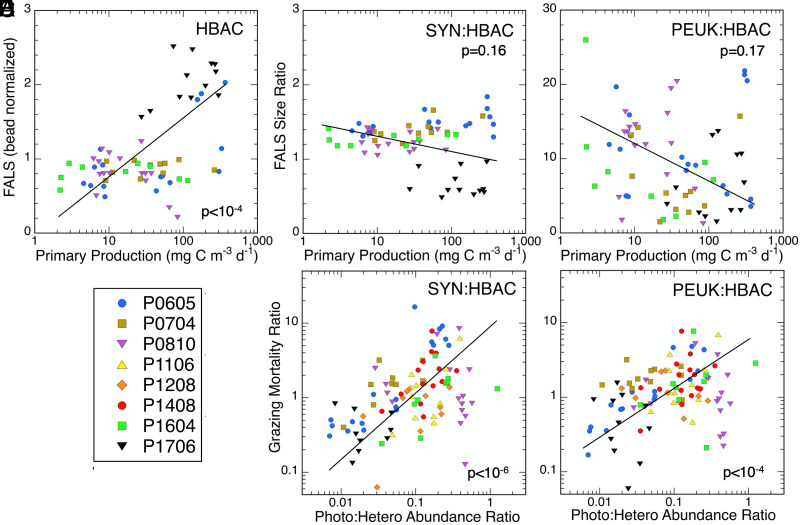
Potential mechanisms to explain observed shifts in grazing mortality on phototrophic picoplankton (SYN and PEUK) relative to heterotrophic HBAC across trophic gradients in the CCE. (*Upper*) Bead-normalized FALS used as an index of HBAC cell size (panel *A*) and mean size ratios of SYN:HBAC and PEUK:HBAC (panels *B* and *C*). (*Lower*) Relationships of grazing mortality ratios SYN:HBAC (panel *D*) and PEUK:HBAC (panel *E*) with relative prey abundances of phototrophic and heterotrophic cells. Full regression statistics are in *SI Appendix*, Table S1.

The simplest explanation for the current results would be if HBAC cells were much larger in the high PP growth environment, greatly increasing their vulnerability to size-selective grazers relative to picophytoplankton. Using bead-normalized forward angle light scatter (FALS) as an index of relative size (34) for the five cruises where we have such data, HBAC cells do in fact show evidence of a size increase at higher PP (Fig. 4*A*). However, the relative size (FALS) ratios of SYN:HBAC and PEUK:HBAC do not indicate a significantly altered size structure for alternate prey (Fig. 4 *B* and *C*). Additionally, since flagellate grazer clearance rates scale with cell radius (35) while FALS scales only to radius^0.55^ (36), cell size variability is insufficient to explain the order of magnitude differences in grazing mortality ratios observed (Fig. 3*F*). We thus reject cell size variability as the main explanation for shifting mortality impacts.

The second mechanism considers changes in grazer behavior or selection for different assemblages of grazing specialists in response to changes in prey characteristics over the trophic gradient. For example, switching behaviors of protistan grazers (37) analogous to those observed for some metazoan zooplankton (38, 39) might involve perceived quality differences between heterotrophic and phototrophic prey and active frequency-reinforced selection based on their relative abundances. For our data, mortality ratios of SYN:HBAC and PEUK:HBAC show strong relationships to the phototroph:heterotroph (P:H) ratio (Fig. 4 *D* and *E*) consistent with switching, or equally with environmental selection of grazer specialists that respond to P:H variability. We cannot rule out however that these relationships simply arise from strong covariability of P:H with PP due mainly to the pronounced decline of PRO in cold productive waters. For grazer switching or highly selective behaviors to be operative at the community level, substantial portions of the assemblages would need to perceive and respond to relative prey qualities similarly. While this may seem unlikely for diverse grazer assemblages, variability in the relative nutritional qualities of HBAC and picophytoplankton prey (stoichiometry, lipids, vitamins, or other factors) could underlie a fitness mechanism that selects for grazer assemblages that match and best utilize the prey resources available (40).

The third hypothesis considers picoplankton populations with vastly different vulnerabilities to protistan grazers independent of size. Given laboratory evidence for significant SYN strain differences in flagellate grazing vulnerability (41, 42), part of the large shift in SYN:HBAC mortality might be explained by selection of SYN defensive traits in high PP waters. It seems more likely, however, that mortality variability would arise principally from the diverse phylogenies, morphotypes, behaviors, and biogeochemical functions of HBAC, rather than variability within a single genus of cyanobacteria. We note, for example, that SYN clade dominance (clades I and IV) and their proportions remained similar in both low (offshore) and high PP (onshore) habitats during P1604 (24) while SYN:HBAC mortality for P1604 showed the same large shifts as other cruises (Fig. 3*F*). For the same cruise, HBAC community composition varied far more extensively, with SAR11 declining from 30 to 7% of the HBAC assemblage from offshore to inshore sampling sites, while Flavobacteria increased >460 fold (200 to >10^5^ cells mL^−1^), OM60 Proteobacteria increased >210 fold (1,600 to >3.3*10^5^ cells mL^−1^), and *Roseobacter* increased >38 fold (12,900 to >4.9*10^5^ cells mL^−1^) (24). Cell surface hydrophobicity and motility are among the factors that can increase bacterial vulnerability to flagellate grazers by factors of 2 or more (43, 44). The notably hydrophilic SAR11, for example, is retained by the fine filters of appendicularians at up to order-of-magnitude lower rates compared to β and γ Proteobacteria with more hydrophobic “sticky” surfaces (45). We posit that HBAC traits that allow certain cell types to achieve high growth rates in enriched substrate waters are balanced by tradeoffs in grazer vulnerability. If so, rate studies aimed at resolving species-level dynamics are likely to reveal heretofore unimagined variability of microbial growth and grazing across ocean productivity gradients.

### Implications for Theory and Models.

Regardless of the specific mechanism, large shifts in relative grazing mortalities on co-occurring microbes challenge the long-held view that protistan grazing mainly determines overall biomass of microbial communities while viruses uniquely regulate diversity by “killing the winners” (17–19). Because the dilution technique used for our experiments does not measure the component of microbial growth lost to viral lysis (46, 47), our mortality results are attributable to grazing only. In high PP waters, successful fast-growing HBAC are clearly punished by compensatory high grazing impacts not experienced by slower-growing phototrophs. For HBAC, this group-level kill-the-winner phenomenon accounts for more than order-of-magnitude variability in mortality rates compared to a common prey type (SYN) over the trophic richness gradient, implying even higher range ratios for extreme HBAC taxa relative to the group mean. Similar to the suggested role for viral lysis, these shifts in relative grazing promote microbial community diversity by selecting against the growth-rate winners, in the present case allowing SYN and PEUK populations to persist in PP-enriched waters despite growth rates substantially less than the average HBAC. One important difference is that the portion of microbial growth lost to viral lysis represents a net energy drain shunted mainly to a closed loop of dissolved organic cycling (48) while a portion of grazed mortality remains available to larger consumers via the trophic hierarchy, often becoming a main source of zooplankton nutrition in oligotrophic habitats (49). These two mortality processes thus have some overlapping functions in production utilization and diversity maintenance but are sufficiently different in food web implications to require separate understanding.

Given the general paucity of comparative microbial grazing rate data for marine systems, it is not surprising that model representations of mortality processes are overly simplistic. Models designed to examine the general interplay of bottom–up and top–down processes in shaping size structure of whole plankton communities typically ignore microbial interactions entirely or parameterize only its nutrient remineralization function (50). Community self-assembly and niche partitioning models both highlight the physiological capabilities of competing ecotypes as the main determinant of distributional outcomes (5, 51, 52), with little consideration of mortality variability among ecotypes or environments. Here, we note that dynamic several-fold variability in microbial mortality pressures that scales with primary productivity might provide insights or explanations for distributional patterns, especially in coastal environments, that are poorly understood or that have previously been ascribed solely to physiological differences. Grazing functions that selectively impact faster-growing populations should substantially alter source population contributions to carbon and energy flows but not necessarily require massive change in model complexity if tradeoffs in growth advantages and grazing vulnerability are rapidly enforced and self-correcting. A judicious first step might be to test alternate model constructs to see what will achieve the best results in systems, like the CCE, with sufficient data on both distributional patterns and process rates.

## Materials and Methods

### Experimental Setup.

On each of eight CCE Process cruises, we conducted multiday quasi-Lagrangian experiments during which we sampled and measured processes on a consistent daily schedule following a satellite-tracked free-drifting array (20). The drift array (Pacific Gyre, San Diego) consisted of a surface float, a 3-m drogue centered at 15 m, coated wire with stainless-steel attachment rings for hanging net bags of bottles for in situ incubations, and a separately attached smaller float with iridium transmission (10-min position frequency) and a nighttime strobe light.

For each experiment, we collected seawater from Niskin bottles on early-morning Conductivity-Temperature-Depth (CTD) hydrocasts (~02:00 local time) at 6 to 8 depths spanning the euphotic zone. For each depth, we prepared a two-treatment dilution experiment (53, 54), with one polycarbonate bottle (2.7 L) containing unfiltered seawater (100%) and the second (diluted) bottle consisting of ~33% whole seawater with filtered water from the same depth. Seawater was filtered directly from the Niskin bottles using a peristaltic pump, silicone tubing, and in-line 0.1-µm Suporcap filter capsules (Pall Acropak) that had previously been acid rinsed and soaked (10% trace metal grade HCl, several hours before use). Dilution bottles (all bottles also 10% HCl rinsed and soaked for the entire period between uses) were first given a measured volume of filtered water and then filled gently to the top with unscreened water from the Niskin bottles to avoid physical damage to fragile protists. Consistent with previous studies, nutrients were not added to the incubation bottles because additions have been found to suppress grazing in oligotrophic waters (55) and ambient concentrations should not limit picophytoplankton growth in productive coastal waters. Each filled bottle was subsampled for flow cytometry (FCM) analysis (1 to 2 mL) for initial microbial concentrations. In addition to the dilution experiments, triplicate 250-mL polycarbonate bottles and one 250-mL “dark” bottle were filled with water from each depth and spiked with H^14^CO_3_^−^ to measure net PP.

All bottles (dilution plus ^14^C-PP) were placed in coarse net bags, attached to the line below the drifter float and incubated in situ for 24 h at the depth of collection. For back-to-back daily experiments, the new experiment was set up in net bags on deck before hand recovery of the drifter. The previous day’s experiments were then removed, the new experiments attached, and the drifter redeployed—a process that took 10 to 15 min while the ship maintained position. Sampling for daily experiments was done in close proximity (~100 m) to the drifter position, and all recovery and deployments were carried out before sunrise. Upon recovery, the bottles were sampled for assessments of community composition (final FCM samples) and ^14^C uptake as described below.

### Population Abundances.

Samples for FCM analysis of HBAC, PRO, SYN, and PEUK populations were preserved with 0.5% paraformaldehyde (final concentration) and flash-frozen in liquid nitrogen. On shore, the samples were stored at −80 °C and then thawed in batches and stained with Hoechst 33342 (1 µg mL^−1^, final concentration) immediately prior to analysis (56). The analyses were done using a Beckman-Coulter Altra flow cytometer equipped with a Harvard Apparatus syringe pump to quantify volume sampled and two argon ion lasers tuned to yellow-green (UV or YG) (200 mW) and 488 nm (1 W) excitation. Fluorescence signals were collected using filters for Hoechst-bound DNA, phycoerythrin, and chlorophyll, all normalized to internal bead standards of 0.5- and 1.0-µm YG polystyrene beads. Listmode files were analyzed with FlowJo software to define populations based on DNA signal (all cells), absence of photosynthetic pigments (HBAC), Chl*a* presence (PRO, SYN, and PEUK), phycoerythrin (SYN), and FALS (relative size). Further methodological details are provided as *SI Appendix*.

### Experimental Rate Determinations.

For each dilution experiment, net rates of population growth from initial and final FCM samples in diluted (k_d_) and undiluted (k) treatments were used to compute microzooplankton grazing mortality (m, d^−1^) and instantaneous growth (µ, d^−1^) as: m = (k_d_ − k)/(1 − D) and µ = k + m, where D is the mean measured dilution factor from initial FCM subsamples of the dilute treatment bottles (53). For each station (=drift array day), incubations conducted in the upper half of the experimental depths were treated as independent rate assessments for the upper euphotic zone, where rates are highest and not light-limited. On average, these rate determinations came from the upper 26.8 m, corresponding closely to the 0-30 m depth range for data from which the EML hypothesis derives (7). All results are presented as mean rates ± SE uncertainties (±SEM) for the upper euphotic zone. Regression analyses were done using R *lmodel2* (version 1.7-3) and protocols (57). For log-scale regressions of mortality or mortality ratios (e.g., Fig. 3), the few rate estimates with negative values (*SI Appendix*, Table S2) were given a low positive value of 0.01. This had no effect on regression significance compared to linear-scale analyses of the same data including negative values.

The process of filtering water to prepare dilution experiments has the potential to release dissolved organics that could bias rate estimates for HBAC, especially in experiments with rich coastal bloom waters with high plankton biomass (58, 59). Relevant to this possible artifact, we found no evidence for a systematic bias between rich and poor waters when comparing dilution-based estimates of bacterial carbon production to standard ^3^H-leucine rates for three of the CCE Process cruises (P1408, P1604, and P1708). For cell carbon conversions informed by size variability from FCM, the two bacterial production estimates give a 1:1 regression relationship across the CCE productivity range (60).

### Associated Environmental Measurements.

Temperature measurements (CTD sensors) and subsamples for Chl*a* and nutrient analyses were taken for all depths on the casts for experimental setup. Chl*a* samples (250 mL) were filtered onto GF/F filters and extracted with 90% acetone at −4 °C for 24 h. Extracted samples were quantified on shipboard with a calibrated Turner Designs model 10 fluorometer. Nutrient samples were filtered through in-line 0.1-µm Suporcap capsules, frozen at −20 °C, and subsequently analyzed in the laboratory against prepared standards by continuous-flow Autoanalyzer or Flow Injection techniques. The contents of PP incubation bottles were filtered through GF/F filters and fumed with HCl to remove inorganic ^14^C. After addition of scintillation cocktail, beta decays were detected with a Beckman–Coulter scintillation counter. Dark bottle uptake was subtracted from light bottle uptake to correct for nonphotosynthetic carbon uptake or adsorption onto particles.

## Supplementary Material

Appendix 01 (PDF)

## Data Availability

All cruise data for CCE Process cruises, including hydrography, nutrients, PP, and population abundances, are publicly available at https://oceaninformatics.ucsd.edu/datazoo/catalogs/ccelter/datasets (61). Mean values and uncertainties (SEM) for environmental variables and population cell abundances are tabulated in *SI Appendix*, Table S2. Mean daily (±SEM) growth and grazing mortality rates of HBAC, PRO, SYN, and PEUK populations are tabulated in *SI Appendix*, Table S3. All other data are included in the article and/or *SI Appendix*.
