# Supplementary material for: Coexisting picoplankton experience different relative grazing pressures across an ocean productivity gradient
Source: Proc Natl Acad Sci U S A. 2023 Oct 23;120(44):e2220771120. doi: 10.1073/pnas.2220771120 (PMC10622918; doi:10.1073/pnas.2220771120)
Supplement: Supplementary file 1 — Appendix 01 (PDF) [file pnas.2220771120.sapp.pdf]

**SUPPORTING INFORMATION**

# Coexisting picoplankton experience different relative grazing pressures across an ocean productivity gradient

Michael R. Landry[a*], Michael R. Stukel[b], Karen E. Selph[c], Ralf Goericke[a]

[a]Scripps Institution of Oceanography, University of California at San Diego, La Jolla, CA 92093;
[b]Earth, Ocean, and Atmospheric Science Department, Florida State University, Tallahassee, FL 32306;
[c]Department of Oceanography, University of Hawai'i at Manoa, Honolulu, HI 96822, USA

**Corresponding author*

***Abstract***

Picophytoplankton populations (*Prochlorococcus*, *Synechococcus* and picoeukaryotes) are dominant primary producers in the open ocean and projected to become more important with climate change. Their fates can vary, however, with microbial food web complexities. In the California Current Ecosystem (CCE), picophytoplankton biomass and abundance peak in waters of intermediate productivity and decrease at higher production. Using experimental data from eight cruises crossing the pronounced CCE trophic gradient, we tested the hypothesis that these declines are driven by intensified grazing on heterotrophic bacteria passed to similarly sized picophytoplankton via shared predators. Results confirm previously observed distributions as well as significant increases in bacterial abundance, cell growth and grazing mortality with primary production. Mortalities of picophytoplankton, however, diverge from the bacterial mortality trend such that relative grazing rates on *Synechococcus* compared to heterotrophic bacteria decline by 12-fold between low and high productivity waters. The large shifts in mortality rate ratios for coexisting populations are not explained by size variability, but rather suggest high selectivity of grazer assemblages or tightly coupled tradeoffs in microbial growth advantages and grazing vulnerabilities. These findings challenge the long-held view that protistan grazing mainly determines overall biomass of microbial communities while viruses uniquely regulate diversity by "killing the winners".

**Table S1.** Regression statistics for plotted variables in Figs. 1-4. A = intercept; B = slope; R = correlation coefficient; p = significance of regression slope B. PP = primary production; P:H = phototroph:heterotroph ratio; Mort = grazing mortality; FALS = forward angle light scatter (cell size proxy). Font colors (black, red, blue) correspond to figure regression lines. Formats for exponential (Expon), logarithmic (Log) and Power functions are $Y=A*10^{B*X}$, $Y=A+B*\log X$ and $Y=A*X^B$, respectively. Regression models are Ordinary Least Squares (OLS, Model 1) and Reduced Major Axis (RMA, Model 2). Values of $p \le 0.05$ are considered significant.

| Fig | Y-variable | X-variable | Function | Model | A | B | R | p |
|---|---|---|---|---|---|---|---|---|
| 1a | Nitrate | Temp | Expon | OLS | 1.89e+5 | -0.367 | 0.798 | 1.71e-23 |
| 1b | PP | Nitrate | Power | OLS | 30.8 | 0.581 | 0.819 | 1.36e-25 |
| 1c | PP | Chl*a* | Power | OLS | 35.2 | 0.951 | 0.871 | 1.07e-32 |
| 2a | HBAC Abund | PP<100 | Power | RMA | 3.81e+5 | 0.361 | 0.436 | 2.01e-4 |
| 2a | HBAC Abund | PP>1 | Power | RMA | 3.28e+5 | 0.355 | 0.347 | 3.73e-3 |
| 2b | PRO Abund | PP<10 | Power | RMA | 1.59e+5 | -0.199 | -0.072 | 0.687 |
| 2c | SYN Abund | PP<100 | Power | RMA | 1.18e+3 | 1.13 | 0.483 | 3.06e-5 |
| 2c | SYN Abund | PP>10 | Power | RMA | 1.46e+6 | -1.10 | -0.481 | 3.28e-5 |
| 2d | PEUK Abund | PP<100 | Power | RMA | 1.14e+3 | 0.939 | 0.810 | 6.15e-17 |
| 2d | PEUK Abund | PP>10 | Power | RMA | 2.10e+4 | -0.010 | -0.008 | 0.948 |
| 2b | PEUK Abund | PP>100 | Power | RMA | 1.03e+8 | -1.62 | -0.454 | 0.067 |
| 2e | HBAC Growth | PP | Log | RMA | -0.119 | 0.394 | 0.592 | 5.57e-11 |
| 2f | PRO Growth | PP | Log | RMA | 0.298 | 0.179 | 0.124 | 0.339 |
| 2g | SYN Growth | PP | Log | RMA | 0.536 | -0.091 | -0.157 | 0.116 |
| 2h | PEUK Growth | PP | Log | RMA | 0.468 | -0.022 | -0.034 | 0.735 |
| 3a | HBAC Graz Mort | PP | Power | RMA | 0.098 | 0.319 | 0.426 | 8.18e-6 |
| 3b | PRO Graz Mort | PP | Power | RMA | 7.14e-2 | 0.544 | 0.161 | 0.214 |
| 3c | SYN Graz Mort | PP | Power | RMA | 1.14 | -0.483 | 0.437 | 4.47e-6 |
| 3d | PEUK Graz Mort | PP | Power | RMA | 0.466 | -0.149 | -0.182 | 0.067 |
| 3e | PRO:HBAC Mort | PP | Power | RMA | 1.11 | -0.019 | -0.006 | 0.964 |
| 3f | SYN:HBAC Mort | PP | Power | RMA | 8.33 | -0.698 | -0.606 | 1.44e-11 |
| 3g | PEUK:HBAC Mort | PP | Power | RMA | 4.90 | -0.480 | -0.496 | 1.13e-7 |
| 4a | HBAC FALS | PP | Log | RMA | -0.077 | 0.817 | 0.487 | 3.38e-5 |
| 4b | SYN:HBAC FALS | PP | Log | RMA | 1.58 | -0.230 | 0.176 | 0.156 |
| 4c | PEUK:HBAC FALS | PP | Log | RMA | 17.3 | -5.13 | -0.171 | 0.169 |
| 4d | SYN:HBAC Mort | P:H Ratio | Power | RMA | 8.61 | 0.887 | 0.543 | 3.84e-9 |
| 4e | PEUK:HBAC Mort | P:H Ratio | Power | RMA | 5.19 | 0.623 | 0.406 | 2.21e-5 |

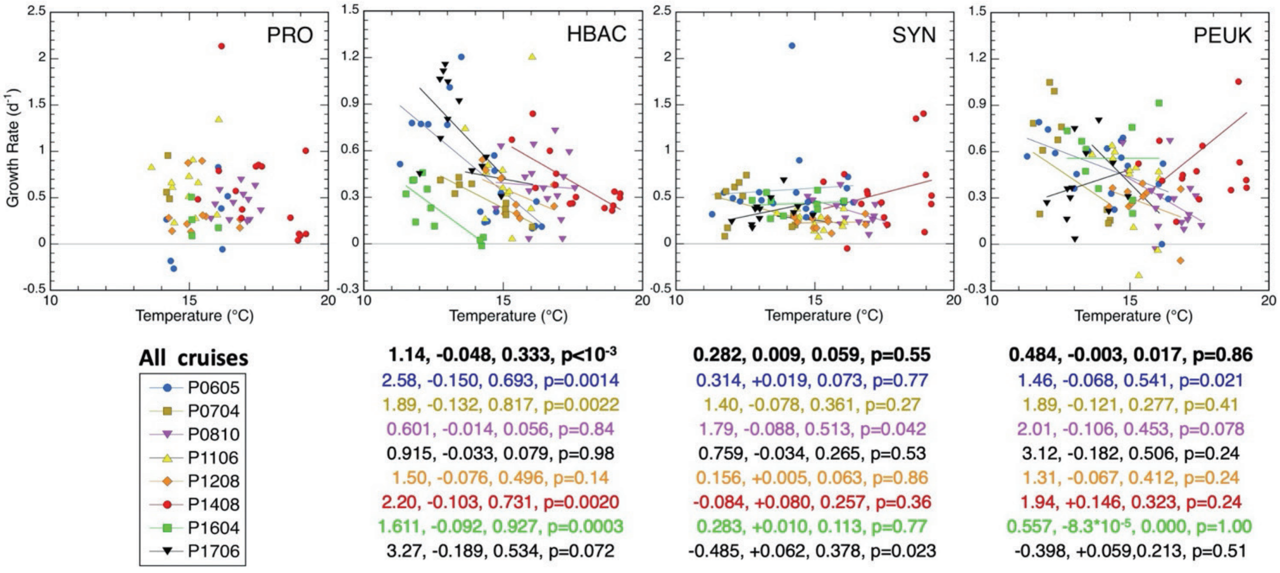


**Figure S1.** Relationships of picoplankton growth rates to upper-euphotic-zone temperatures during CCE Process cruises. HBAC = heterotrophic bacteria; PRO = *Prochlorococcus*; SYN = *Synechococcus*; PEUK = photosynthetic picoeukaryotes. Regression relationships (Ordinary Least Squares because the X-axis variable *temperature* is precisely measured) are color coded to the cruise abbreviation legend and given in the format: regression intercept, regression slope, multiple R correlation coefficient, and significance (p) of regression slope. Regression statistics for all cruises combined are given in bold font, but those regression lines are not plotted.

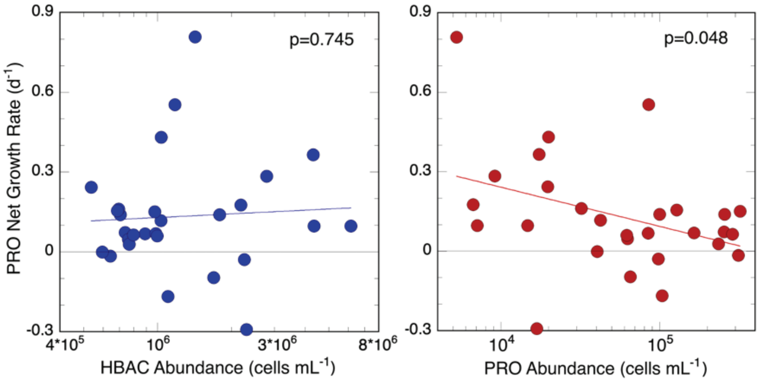


**Figure S2.** Relationships between net growth rate of PRO and population abundances of HBAC and PRO for experiments conducted at intermediate values of primary production (10<PP<100 mg C $m^{-3}$ $d^{-1}$).

## Flow Cytometry Methods

Flow cytometry (FCM) samples were analyzed using a Beckman Coulter EPICS Altra flow cytometer with colinear dual laser excitation (488 nm,1 W; UV, 200 mW) and a Harvard apparatus syringe pump for volumetric sample delivery of 100 µL at 50 µl $min^{-1}$. Discrete populations were enumerated on the basis of chlorophyll *a* (red fluorescence, 680 nm BP filter), phycoerythrin (orange fluorescence, 575 nm BP filter), DNA (blue fluorescence, 450 nm BP filter), forward angle light scatter, (FALS; ND1 blocking and 488 nm BP filters) and 90° light scatter (488 nm BP filter) signatures. Data were generated as listmode files (FCS 2.0 format) acquired from the flow cytometer using Expo32 software (Beckman Coulter).

Population designations and their associated fluorescence and scatter parameters were generated from the listmode files using FlowJo software (Tree Star, Inc.) and exported to spreadsheet format (e.g., Excel files). Several successive steps were used to define populations (Fig. S3). Phycoerythrin-bearing *Synechococcus* (SYN) were separated from all other cells (Fig. S3a). Heterotrophic bacteria (HBAC) were distinguished from phytoplankton by absence of pigment fluorescence (Fig. S3b) and low DNA fluorescence (Fig. S3d). *Prochlorococcus* (PRO) and photosynthetic eukaryotes (PEUK) were separated from each other by their characteristic DNA, chlorophyll and light scatter signatures (Fig S3b & c). PEUK (mostly <2 µm pico- and nano-sized cells due to the small volume analyzed) generally had higher light scatter per cell than prokaryotes, although some PEUK had similar light scatter but higher DNA fluorescence. Note that light scatter signatures are only proportional to cell size, since scattered light is generated based on an object's size, shape, and internal structure or refractive index (Robertson and Button 1989; Olson et al. 1989; Olson et al. 1993).

In addition, a mixture of fluorescent polystyrene bead standards was analyzed on each run day to obtain mean estimates of scatter and fluorescence for normalization of each signal and to check instrument alignment. "Normalization" refers to dividing the population mean scatter or fluorescence parameter by the corresponding mean parameter of the beads, which provides a stable basis for comparison of population cell characteristics within and between runs. The beads used were 0.5 µm yellow-green beads, 0.5 µm UV beads, and 1 µm yellow-green beads (Polysciences, Inc). Yellow-green beads are optimally excited by the 488 nm laser and emit in all visible wavelengths, whereas UV

beads are excited mainly by the UV laser and emit in the blue (450 nm) range. Since most cells analyzed were ≤2-µm diameter (i.e., picoplankton), the 0.5 µm yellow-green bead signals were used to normalize all light scatter, orange and red fluorescence signals for all populations. The 0.5 µm UV beads were used to normalize blue fluorescence from Hoechst-bound DNA in all populations.

We assessed potential variability in relative cell sizes of phototrophic and heterotrophic bacteria across the CCE production gradient using the ratios of bead-normalized values of forward angle light scatter (FALS) of SYN or PEUK compared to HBAC (i.e., $FALS_{SYN}$:$FALS_{HBAC}$) for populations analyzed in the same samples (Landry et al. 2003, 2022). This derives from the near-linear relationship between FALS and Mie scattering cross section for cells in the submicron-micron size range (DuRand and Olson 1996).

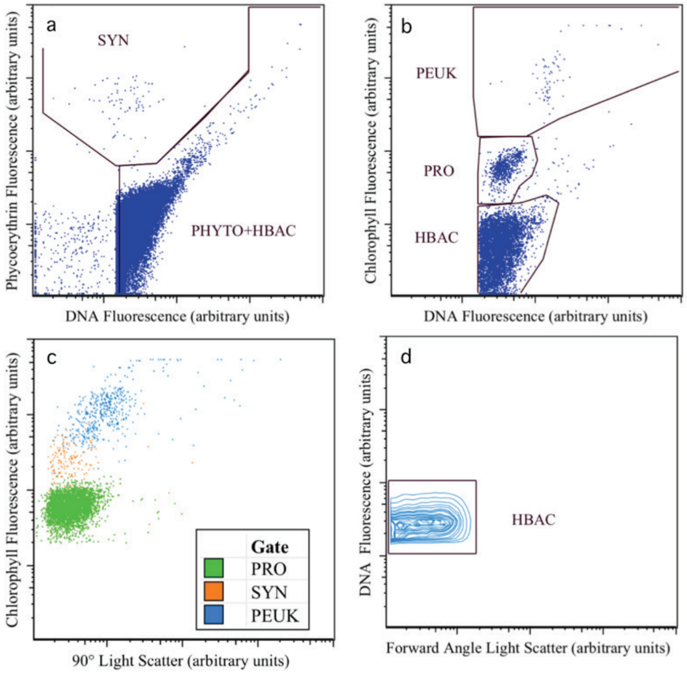


Figure S3. Listmode (FCS 2.0) data were analyzed in FlowJo software to define HBAC and picophytoplankton populations PRO, SYN and PEUK. a) First, phycoerythrin fluorescence was plotted as a function of DNA fluorescence for all events, to separate SYN from the rest of the cells (PHYTO+HBAC). b) Next, the PHYTO+HBAC cells were separated into HBAC, PRO and PEUK by plotting chlorophyll fluorescence as a function of DNA fluorescence. c) All phytoplankton populations (PRO, SYN, and PEUK) are shown as a function of chlorophyll vs. 90° light scatter. d) HBAC are shown as a function of DNA fluorescence vs. forward angle light scatter.

## References

DuRand MD, Olson RJ. (1996) Contributions of phytoplankton light scattering and cell concentration changes to diel variations in beam attenuation in the equatorial Pacific from flow cytometric measurements of pico-, ultra and nanoplankton. *Deep-Sea Res II* 43:891–906.

Landry MR, Brown SR, Neveux J, Dupouy C, Blanchot J, Christensen S, Bidigare RR. (2003) Phytoplankton growth and microzooplankton grazing in high-nutrient, low-chlorophyll waters of the equatorial Pacific: Community and taxon-specific rate assessments from pigment and flow cytometric analyses. *J Geophys Res* 108(C12), 8142, doi:10.1029/2000JC000744.

Landry MR, Selph KE, Hood RR, Davies CH, Beckley LE. (2022). Low temperature sensitivity of picophytoplankton P:B ratios and growth rates across a natural 10°C temperature gradient in the oligotrophic Indian Ocean. *Limnol Oceanogr Lett* **7**: 112–121.

Olson RJ, Zettler ER, Anderson OK (1989) Discrimination of eukaryotic phytoplankton cell types from light scatter and autofluorescence properties measured by flow cytometry. *Cytometry* 10:636-643.

Olson RJ, Zettler ER, DuRand MD (1993) Phytoplankton analysis using flow cytometry. Handbook of Methods in Aquatic Microbial Ecology, CRC Press. pp. 187-197.

Robertson BR, Button DK (1989) Characterizing aquatic bacteria according to population, cell size, and apparent DNA content by flow cytometry. *Cytometry* 10:70-76.

**Table S2.** Environmental variables (temperature, nitrate, chlorophyll a), rates of primary production rates (PP) and cell abundances of heterotrophic bacteria (HBAC), *Prochlorococcus* (PRO), *Synechococcus* (SYN) and photosynthetic picoeukaryotes (PEUK) in the California Current Ecosystem. Data are averaged for experiments in the upper euphotic zone. Uncertainties are standard errors of mean (SEM) values.

| Cruise | Date | Lat (°N) | Long (°W) | Temp (°C) | Nitrate (µM) | Chl*a* (mg $m^{-3}$) | PP (mg C $m^{-3}$ $d^{-1}$) | HBAC ($10^3$ cells $mL^{-1}$) | PRO ($10^3$ cells $mL^{-1}$) | SYN ($10^3$ cells $mL^{-1}$) | PEUK ($10^3$ cells $mL^{-1}$) |
|---|---|---|---|---|---|---|---|---|---|---|---|
| P0605 | 11May2006 | 34.33 | -120.80 | 11.3 ± 0.0 | 13.53 ± 0.04 | 2.92 ± 0.09 | 155.3 ± 33.2 | 1,066 ± 7 | - | 5.75 ± 0.16 | 7.34 ± 0.22 |
| P0605 | 12May2006 | 34.29 | -120.84 | 11.7 ± 0.0 | 11.23 ± 0.16 | 5.47 ± 0.15 | 367.2 ± 120.6 | 1,460 ± 28 | - | 10.28 ± 0.06 | 19.17 ± 0.67 |
| P0605 | 13May2006 | 34.29 | -120.98 | 12.1 ± 0.0 | 9.52 ± 0.60 | 5.86 ± 0.12 | 366.7 ± 84.0 | 1,948 ± 26 | - | 14.17 ± 0.46 | 23.20 ± 1.04 |
| P0605 | 14May2006 | 34.26 | -121.06 | 12.3 ± 0.0 | 7.54 ± 1.26 | 6.50 ± 0.14 | 175.3 ± 57.0 | 2,478 ± 27 | - | 13.88 ± 0.21 | 21.31 ± 0.67 |
| P0605 | 17May2006 | 33.50 | -122.11 | 14.2 ± 0.1 | 0.10 ± 0.01 | 0.15 ± 0.01 | 7.7 ± 0.5 | 888 ± 33 | 68.0 ± 6.0 | 12.19 ± 2.94 | 6.18 ± 0.32 |
| P0605 | 19May2006 | 33.62 | -122.13 | 14.3 ± 0.2 | 0.11 ± 0.01 | 0.13 ± 0.02 | 8.8 ± 0.4 | 738 ± 51 | 152.8 ± 10.1 | 27.06 ± 11.19 | 6.78 ± 0.64 |
| P0605 | 20May2006 | 33.49 | -122.09 | 14.4 ± 0.5 | 0.05 ± 0.01 | 0.13 ± 0.02 | 8.2 ± 0.5 | 790 ± 22 | 110.6 ± 11.4 | 13.39 ± 4.16 | 5.67 ± 0.23 |
| P0605 | 22May2006 | 34.63 | -120.77 | 13.1 ± 0.0 | 3.67 ± 1.52 | 5.86 ± 0.69 | 303.0 ± 121.1 | 2,142 ± 208 | - | 7.33 ± 0.55 | 9.05 ± 1.13 |
| P0605 | 23May2006 | 34.75 | -120.77 | 13.0 ± 0.0 | 3.87 ± 2.52 | 5.25 ± 1.67 | 302.9 ± 121.4 | 2,150 ± 380 | - | 6.17 ± 1.07 | 9.63 ± 2.50 |
| P0605 | 24May2006 | 34.86 | -120.80 | 13.5 ± 0.0 | 1.41 ± 0.08 | 6.21 ± 0.14 | 328.6 ± 88.5 | 2,005 ± 96 | - | 6.93 ± 0.35 | 7.11 ± 0.40 |
| P0605 | 26May2006 | 34.05 | -121.30 | 14.9 ± 0.0 | 0.78 ± 0.02 | 0.73 ± 0.01 | 66.3 ± 7.8 | 1,033 ± 7 | - | 29.11 ± 0.21 | 26.98 ± 0.28 |
| P0605 | 27May2006 | 34.02 | -121.43 | 14.7 ± 0.0 | 0.84 ± 0.01 | 0.97 ± 0.02 | 49.7 ± 5.1 | 973 ± 4 | - | 28.43 ± 0.31 | 24.86 ± 0.25 |
| P0605 | 28May2006 | 33.96 | -121.57 | 14.7 ± 0.0 | 0.91 ± 0.01 | 0.98 ± 0.01 | 52.5 ± 5.1 | 914 ± 5 | - | 27.74 ± 0.59 | 22.75 ± 0.26 |
| P0605 | 30May2006 | 33.87 | -121.72 | 14.7 ± 0.0 | 0.89 ± 0.02 | 0.61 ± 0.01 | 43.3 ± 5.8 | 1,021 ± 3 | - | 12.45 ± 0.12 | 15.41 ± 0.26 |
| P0605 | 01June2006 | 32.85 | -124.00 | 16.0 ± 0.0 | 0.08 ± 0.03 | 0.11 ± 0.00 | 5.7 ± 0.5 | 761 ± 18 | 150.2 ± 18.8 | 5.91 ± 0.30 | 2.94 ± 0.23 |
| P0605 | 02June2006 | 32.78 | -124.09 | 16.4 ± 0.1 | 0.14 ± 0.02 | 0.10 ± 0.00 | 4.5 ± 0.4 | 763 ± 3 | 125.2 ± 6.0 | 4.30 ± 0.08 | 2.89 ± 0.13 |
| P0605 | 03June2006 | 32.67 | -124.19 | 16.1 ± 0.2 | 0.12 ± 0.02 | 0.10 ± 0.01 | 8.5 ± 0.9 | 808 ± 21 | 175.6 ± 7.9 | 5.01 ± 0.49 | 3.58 ± 0.32 |
| P0605 | 04June2006 | 32.49 | -124.25 | 16.2 ± 0.2 | 0.14 ± 0.01 | 0.10 ± 0.00 | 6.4 ± 0.4 | 710 ± 15 | 191.1 ± 11.5 | 3.47 ± 0.08 | 2.62 ± 0.14 |
| P0704 | 05Apr2007 | 34.28 | -120.91 | 12.1 ± 0.0 | 8.08 ± 0.03 | 1.60 ± 0.07 | 48.5 ± 4.3 | 1,037 ± 30 | - | 12.69 ± 0.08 | 37.06 ± 1.02 |
| P0704 | 06Apr2007 | 34.33 | -120.95 | 12.4 ± 0.0 | 5.43 ± 0.13 | 0.98 ± 0.02 | 21.9 ± 4.1 | 907 ± 14 | - | 12.47 ± 0.27 | 37.05 ± 0.97 |

| | | | | | | | | | | | |
|---|---|---|---|---|---|---|---|---|---|---|---|
| P0704 | 07Apr2007 | 34.36 | -121.05 | 12.3 ± 0.1 | 6.19 ± 0.73 | 0.91 ± 0.05 | 26.6 ± 3.9 | 813 ± 20 | - | 11.94 ± 0.98 | 27.84 ± 2.47 |
| P0704 | 10Apr2007 | 33.55 | -123.16 | 14.3 ± 0.0 | 0.06 ± 0.02 | 0.18 ± 0.00 | 9.0 ± 0.2 | 925 ± 23 | 95.0 ± 3.6 | 14.43 ± 0.87 | 9.31 ± 0.09 |
| P0704 | 11Apr2007 | 33.41 | -123.28 | 14.2 ± 0.0 | 0.10 ± 0.05 | 0.19 ± 0.00 | 9.3 ± 0.5 | 925 ± 15 | 89.5 ± 1.3 | 15.27 ± 0.44 | 9.59 ± 0.28 |
| P0704 | 12Apr2007 | 33.33 | -123.45 | 14.2 ± 0.0 | 0.07 ± 0.01 | 0.22 ± 0.00 | 11.1 ± 0.7 | 889 ± 10 | 84.7 ± 0.8 | 15.73 ± 0.33 | 10.52 ± 0.18 |
| P0704 | 15Apr2007 | 34.22 | -120.62 | 11.8 ± 0.0 | 10.48 ± 0.19 | 3.60 ± 0.13 | 262.7 ± 37.0 | 1,038 ± 21 | - | 4.45 ± 0.11 | 6.79 ± 0.41 |
| P0704 | 16Apr2007 | 34.23 | -121.19 | 11.8 ± 0.0 | 11.10 ± 0.02 | 1.10 ± 0.02 | 87.4 ± 16.2 | 814 ± 2 | - | 5.11 ± 0.10 | 15.32 ± 0.16 |
| P0704 | 17Apr2007 | 33.92 | -121.16 | 11.9 ± 0.2 | 9.53 ± 0.91 | 0.69 ± 0.23 | 56.7 ± 22.1 | 740 ± 26 | - | 4.55 ± 1.21 | 12.54 ± 4.54 |
| P0704 | 18Apr2007 | 33.82 | -121.08 | 11.5 ± 0.5 | 7.87 ± 0.02 | 1.02 ± 0.02 | 53.7 ± 14.9 | 961 ± 10 | - | 5.77 ± 0.11 | 20.40 ± 0.35 |
| P0704 | 19Apr2007 | 33.74 | -121.00 | 12.5 ± 0.0 | 4.41 ± 0.01 | 0.75 ± 0.03 | 35.8 ± 4.4 | 935 ± 16 | - | 7.85 ± 0.19 | 22.50 ± 0.67 |
| P0810 | 04Oct2008 | 34.13 | -120.98 | 17.2 ± 0.1 | 0.21 ± 0.04 | 0.32 ± 0.07 | 10.1 ± 0.8 | 737 ± 78 | 255.3 ± 27.5 | 46.41 ± 12.17 | 6.62 ± 1.34 |
| P0810 | 05Oct2008 | 34.11 | -120.84 | 16.4 ± 0.6 | 0.71 ± 0.40 | 0.46 ± 0.16 | 14.1 ± 1.9 | 643 ± 94 | 313.5 ± 49.4 | 46.29 ± 16.53 | 5.96 ± 1.67 |
| P0810 | 06Oct2008 | 33.97 | -120.77 | 16.9 ± 0.1 | 0.27 ± 0.04 | 0.41 ± 0.03 | 10.2 ± 2.1 | 706 ± 43 | 256.4 ± 35.4 | 60.44 ± 1.58 | 11.10 ± 0.53 |
| P0810 | 07Oct2008 | 33.78 | -120.73 | 16.1 ± 0.4 | 0.46 ± 0.07 | 1.75 ± 0.69 | 27.7 ± 6.7 | 767 ± 13 | 234.7 ± 17.2 | 41.48 ± 8.99 | 12.90 ± 0.65 |
| P0810 | 09Oct2008 | 32.89 | -123.69 | 17.4 ± 0.5 | 0.54 ± 0.03 | 0.23 ± 0.05 | 8.5 ± 0.9 | 374 ± 47 | 142.2 ± 11.9 | 4.35 ± 0.53 | 10.04 ± 0.15 |
| P0810 | 10Oct2008 | 32.80 | -123.66 | 17.6 ± 0.1 | 0.26 ± 0.09 | 0.19 ± 0.01 | 8.0 ± 0.8 | 442 ± 43 | 159.6 ± 5.0 | 4.18 ± 0.04 | 6.21 ± 0.12 |
| P0810 | 12Oct2008 | 32.45 | -123.73 | 16.9 ± 0.0 | 0.25 ± 0.02 | 0.25 ± 0.00 | 7.3 ± 2.2 | 722 ± 26 | 311.9 ± 25.6 | 10.61 ± 0.07 | 10.28 ± 0.28 |
| P0810 | 13Oct2008 | 32.29 | -123.84 | 16.6 ± 0.3 | 0.21 ± 0.03 | 0.27 ± 0.01 | 7.5 ± 2.0 | 656 ± 66 | 322.8 ± 13.0 | 10.81 ± 1.22 | 10.35 ± 1.21 |
| P0810 | 16Oct2008 | 34.02 | -121.76 | 15.8 ± 0.0 | 0.34 ± 0.03 | 0.82 ± 0.01 | 37.1 ± 4.0 | 798 ± 30 | 288.6 ± 5.0 | 36.59 ± 3.79 | 19.48 ± 0.58 |
| P0810 | 17Oct2008 | 34.20 | -121.58 | 15.9 ± 0.1 | 0.14 ± 0.03 | 0.77 ± 0.03 | 31.0 ± 3.3 | 974 ± 40 | 322.37 ± 9.3 | 44.68 ± 0.89 | 21.87 ± 0.42 |
| P0810 | 19Oct2008 | 33.61 | -121.15 | 15.9 ± 0.1 | 0.06 ± 0.01 | 0.83 ± 0.09 | 27.2 ± 7.3 | 1,840 ± 86 | - | 39.42 ± 1.14 | 13.44 ± 0.42 |
| P0810 | 20Oct2008 | 33.61 | -121.16 | 16.0 ± 0.0 | 0.18 ± 0.04 | 0.86 ± 0.04 | 31.3 ± 6.3 | 1,223 ± 17 | - | 35.06 ± 2.14 | 13.78 ± 0.27 |
| P0810 | 22Oct2008 | 32.92 | -120.90 | 14.7 ± 0.0 | 2.63 ± 0.04 | 1.65 ± 0.05 | 83.8 ± 24.5 | 3,306 ± 72 | - | 92.37 ± 1.57 | 45.49 ± 1.26 |
| P0810 | 23Oct2008 | 32.82 | -120.84 | 14.9 ± 0.1 | 2.05 ± 0.10 | 1.74 ± 0.13 | 65.0 ± 14.5 | 2,075 ± 73 | - | 57.70 ± 3.86 | 43.20 ± 1.25 |
| P0810 | 26Oct2008 | 32.60 | -120.56 | 17.1 ± 0.1 | 0.15 ± 0.02 | 0.25 ± 0.04 | 6.2 ± 0.5 | 811 ± 10 | 131.0 ± 3.1 | 16.22 ± 1.71 | 9.28 ± 0.63 |
| P0810 | 27Oct2008 | 32.57 | -120.33 | 17.1 ± 0.0 | 0.19 ± 0.09 | 0.23 ± 0.01 | 6.7 ± 0.7 | 945 ± 12 | 243.9 ± 13.8 | 19.25 ± 0.95 | 8.39 ± 0.37 |
| P1106 | 24June2011 | 34.03 | -121.70 | 14.5 ± 0.0 | 2.94 ± 0.00 | 0.50 ± 0.00 | 22.8 ± 1.0 | 761 ± 32 | 62.8 ± 7.6 | 35.33 ± 0.49 | 33.95 ± 0.44 |
| P1106 | 25June2011 | 33.91 | -121.46 | 14.4 ± 0.0 | 3.08 ± 0.01 | 0.54 ± 0.00 | 19.5 ± 3.1 | 596 ± 16 | 40.4 ± 4.6 | 34.09 ± 0.49 | 30.41 ± 1.94 |
| P1106 | 27June2011 | 33.58 | -121.77 | 15.1 ± 0.0 | 0.02 ± 0.00 | 0.12 ± 0.00 | 4.4 ± 0.2 | 311 ± 7 | 0.3 ± 0.0 | 18.53 ± 0.92 | 8.51 ± 0.32 |
| P1106 | 28June2011 | 33.42 | -121.62 | 15.2 ± 0.0 | 0.03 ± 0.01 | 0.11 ± 0.00 | 4.7 ± 0.2 | 533 ± 57 | 105.6 ± 2.2 | 22.37 ± 0.98 | 8.38 ± 0.41 |

| | | | | | | | | | | | |
|---|---|---|---|---|---|---|---|---|---|---|---|
| P1106 | 30June2011 | 34.11 | -121.65 | 13.6 ± 0.0 | 8.18 ± 0.97 | 0.60 ± 0.08 | 26.8 ± 0.7 | 1,036 ± 59 | 19.9 ± 4.6 | 2.85 ± 0.27 | 27.91 ± 3.72 |
| P1106 | 01July2011 | 34.06 | -121.47 | 14.4 ± 0.0 | 5.94 ± 0.07 | 0.49 ± 0.01 | 33.2 ± 2.1 | 537 ± 27 | 19.7 ± 0.4 | 3.24 ± 0.14 | 23.17 ± 0.37 |
| P1106 | 07July2011 | 33.53 | -121.09 | 15.3 ± 0.1 | 0.72 ± 0.58 | 2.00 ± 0.18 | 49.4 ± 14.3 | 2,258 ± 122 | 98.1 ± 5.5 | 106.0 ± 3.6 | 24.81 ± 0.48 |
| P1106 | 08July2011 | 33.39 | -121.16 | 16.0 ± 0.0 | 0.05 ± 0.01 | 2.73 ± 0.10 | 39.6 ± 13.7 | 1,490 ± 54 | - | 110.8 ± 2.5 | 22.86 ± 0.58 |
| P1106 | 10July2011 | 33.42 | -121.39 | 16.1 ± 0.0 | 0.00 ± 0.00 | 0.12 ± 0.00 | 5.1 ± 0.2 | 391 ± 24 | 135.7 ± 3.4 | 9.04 ± 0.50 | 7.86 ± 0.69 |
| P1106 | 11July2011 | 33.08 | -121.41 | 16.0 ± 0.0 | 0.02 ± 0.00 | 0.12 ± 0.01 | ND | 686 ± 15 | 128.3 ± 4.0 | 8.80 ± 0.23 | 8.78 ± 0.30 |
| P1106 | 13July2011 | 33.52 | -121.11 | 15.0 ± 0.1 | 2.81 ± 0.93 | 0.36 ± 0.04 | 14.2 ± 2.5 | 695 ± 21 | 32.1 ± 2.1 | 14.74 ± 2.66 | 21.71 ± 2.87 |
| P1106 | 14July2011 | 33.07 | -121.26 | 15.0 ± 0.1 | 2.31 ± 0.18 | 0.50 ± 0.01 | 20.4 ± 4.4 | 1,032 ± 62 | 42.4 ± 1.0 | 37.52 ± 1.67 | 39.94 ± 1.32 |
| P1208 | 06Aug2012 | 34.56 | -122.77 | 14.3 ± 0.0 | 2.48 ± 0.05 | 4.09 ± 0.51 | 61.2 ± 20.9 | 4,347 ± 238 | 14.7 ± 1.4 | 85.58 ± 5.48 | 31.76 ± 0.69 |
| P1208 | 07Aug2012 | 34.21 | -122.90 | 14.4 ± 0.1 | 1.73 ± 0.25 | 5.83 ± 0.09 | 72.3 ± 21.2 | 6,144 ± 159 | 7.1 ± 0.2 | 90.42 ± 14.98 | 23.93 ± 1.48 |
| P1208 | 08Aug2012 | 33.85 | -122.96 | 15.0 ± 0.3 | 1.32 ± 0.43 | 0.54 ± 0.00 | 65.1 ± 12.3 | 4,315 ± 116 | 17.4 ± 2.2 | 246.1 ± 52.1 | 26.24 ± 0.36 |
| P1208 | 10Aug2012 | 34.57 | -123.04 | 15.6 ± 0.1 | 0.50 ± 0.03 | 0.36 ± 0.01 | 13.1 ± 1.7 | 984 ± 13 | 164.5 ± 2.3 | 29.40 ± 0.86 | 15.43 ± 0.31 |
| P1208 | 11Aug2012 | 34.09 | -123.26 | 15.1 ± 0.2 | 0.59 ± 0.09 | 0.53 ± 0.07 | 28.0 ± 2.2 | 1,103 ± 54 | 103.7 ± 1.8 | 28.82 ± 2.11 | 16.91 ± 1.07 |
| P1208 | 13Aug2012 | 34.54 | -122.50 | 15.5 ± 0.1 | 0.82 ± 0.05 | 0.96 ± 0.08 | 30.7 ± 6.4 | 2,786 ± 105 | 9.2 ± 0.7 | 203.6 ± 4.2 | 14.48 ± 0.38 |
| P1208 | 14Aug2012 | 34.50 | -122.48 | 15.5 ± 0.2 | 0.85 ± 0.14 | 1.11 ± 0.12 | 33.1 ± 6.4 | 2,189 ± 277 | 6.7 ± 0.3 | 143.6 ± 13.0 | 16.79 ± 0.56 |
| P1208 | 16Aug2012 | 34.54 | -123.27 | 16.8 ± 0.0 | 0.01 ± 0.01 | 0.09 ± 0.00 | 2.8 ± 0.1 | 373 ± 25 | 101.3 ± 3.2 | 2.17 ± 0.08 | 2.55 ± 0.05 |
| P1208 | 17Aug2012 | 34.31 | -123.46 | 16.8 ± 0.0 | 0.02 ± 0.01 | 0.10 ± 0.01 | 3.1 ± 0.1 | 374 ± 10 | 77.4 ± 1.6 | 2.27 ± 0.18 | 3.05 ± 0.15 |
| P1208 | 19Aug2012 | 34.08 | -123.32 | 14.9 ± 0.4 | 1.33 ± 0.40 | 0.66 ± 0.11 | 28.4 ± 9.2 | 997 ± 222 | 62.0 ± 26.0 | 46.93 ± 11.79 | 17.92 ± 2.73 |
| P1408 | 11Aug2014 | 34.51 | -120.77 | 15.3 ± 0.5 | 0.41 ± 0.33 | 1.85 ± 0.07 | 14.0 ± 1.5 | 2,305 ± 157 | 16.9 ± 3.0 | 50.55 ± 8.55 | 14.94 ± 1.88 |
| P1408 | 12Aug2014 | 34.72 | -121.00 | 16.2 ± 0.2 | 0.04 ± 0.01 | 0.60 ± 0.03 | 8.0 ± 0.7 | 1,953 ± 160 | 15.4 ± 2.3 | 93.44 ± 2.46 | 15.92 ± 0.95 |
| P1408 | 13Aug2014 | 34.81 | -121.22 | 16.1 ± 0.5 | 0.17 ± 0.08 | 0.84 ± 0.18 | 14.6 ± 1.4 | 1,423 ± 294 | 5.3 ± 0.3 | 34.38 ± 7.50 | 10.56 ± 1.28 |
| P1408 | 17Aug2014 | 34.27 | -120.82 | 16.7 ± 0.1 | 0.06 ± 0.00 | 0.75 ± 0.04 | 10.8 ± 1.7 | 1,179 ± 103 | 85.3 ± 2.7 | 105.1 ± 2.3 | 11.82 ± 0.51 |
| P1408 | 18Aug2014 | 34.17 | -120.87 | 16.9 ± 0.0 | 0.08 ± 0.01 | 0.79 ± 0.01 | 12.0 ± 2.8 | 1,793 ± 52 | 99.9 ± 2.1 | 110.0 ± 3.8 | 13.02 ± 0.39 |
| P1408 | 19Aug2014 | 34.12 | -120.92 | 16.9 ± 0.0 | 0.07 ± 0.01 | 0.85 ± 0.01 | 11.0 ± 2.3 | 1,697 ± 32 | 65.4 ± 2.2 | 105.7 ± 0.64 | 12.09 ± 0.28 |
| P1408 | 22Aug2014 | 34.39 | -121.39 | 17.5 ± 1.0 | 0.36 ± 0.32 | 0.31 ± 0.11 | 6.8 ± 0.3 | 1,601 ± 37 | 113.9 ± 14.6 | 60.76 ± 1.83 | 11.56 ± 2.67 |
| P1408 | 23Aug2014 | 34.41 | -121.28 | 17.6 ± 0.8 | 0.24 ± 0.17 | 0.37 ± 0.16 | 6.7 ± 1.1 | 1,473 ± 83 | 225.4 ± 37.9 | 67.30 ± 5.21 | 11.48 ± 2.92 |
| P1408 | 24Aug2014 | 34.43 | -121.15 | 17.4 ± 0.9 | 0.28 ± 0.26 | 0.33 ± 0.11 | 6.7 ± 1.1 | 1,283 ± 44 | 133.7 ± 14.0 | 63.98 ± 1.09 | 11.67 ± 3.07 |
| P1408 | 26Aug2014 | 33.52 | -122.56 | 19.0 ± 0.1 | 0.02 ± 0.01 | 0.11 ± 0.00 | 2.2 ± 0.2 | 1,133 ± 23 | 179.1 ± 7.4 | 4.24 ± 0.33 | 4.04 ± 0.25 |
| P1408 | 27Aug2014 | 33.54 | -122.51 | 18.9 ± 0.3 | 0.05 ± 0.00 | 0.11 ± 0.01 | 2.3 ± 0.1 | 1,100 ± 65 | 200.8 ± 7.1 | 3.03 ± 0.61 | 3.17 ± 0.82 |

| | | | | | | | | | | | | |
|---|---|---|---|---|---|---|---|---|---|---|---|---|
| P1408 | 28Aug2014 | 33.60 | -122.44 | 18.6 ± 0.4 | 0.04 ± 0.01 | 0.10 ± 0.01 | 2.0 ± 0.1 | 1,173 ± 21 | 142.3 ± 1.1 | 1.67 ± 0.14 | 3.89 ± 0.38 |
| P1408 | 30Aug2014 | 32.88 | -123.90 | 19.0 ± 0.5 | 0.05 ± 0.01 | 0.10 ± 0.01 | 1.9 ± 0.2 | 728 ± 40 | 254.8 ± 4.6 | 2.51 ± 0.23 | 0.76 ± 0.08 |
| P1408 | 31Aug2014 | 32.84 | -123.87 | 19.2 ± 0.5 | 0.01 ± 0.00 | 0.10 ± 0.01 | 1.8 ± 0.1 | 701 ± 59 | 153.6 ± 5.5 | 2.66 ± 0.05 | 2.27 ± 0.99 |
| P1408 | 01Sept2014 | 32.81 | -123.87 | 19.2 ± 0.6 | 0.04 ± 0.01 | 0.10 ± 0.01 | 2.0 ± 0.1 | 703 ± 94 | 121.1 ± 0.7 | 3.00 ± 0.47 | 4.27 ± 0.58 |
| P1604 | 22Apr2016 | 33.09 | -122.94 | 16.0 ± 0.4 | 0.03 ± 0.01 | 0.11 ± 0.03 | 2.2 ± 0.2 | 271 ± 6 | 327.9 ± 25.4 | 7.29 ± 4.19 | 2.34 ± 0.26 |
| P1604 | 29Apr2016 | 33.59 | -122.21 | 15.1 ± 0.0 | 0.10 ± 0.02 | 0.15 ± 0.01 | 4.4 ± 1.4 | 632 ± 22 | 155.3 ± 4.4 | 14.60 ± 2.38 | 2.36 ± 0.17 |
| P1604 | 30Apr2016 | 33.52 | -122.06 | 15.1 ± 0.0 | 0.09 ± 0.03 | 0.14 ± 0.01 | 2.9 ± 0.3 | 585 ± 18 | 135.1 ± 3.1 | 10.03 ± 1.16 | 2.91 ± 0.20 |
| P1604 | 01May2016 | 33.47 | -121.97 | 15.1 ± 0.0 | 0.05 ± 0.02 | 0.15 ± 0.01 | 2.3 ± 0.5 | 562 ± 13 | 139.5 ± 5.6 | 8.93 ± 0.42 | 2.60 ± 0.17 |
| P1604 | 03May2016 | 34.80 | -121.28 | 12.8 ± 0.2 | 6.69 ± 0.89 | 0.85 ± 0.01 | 16.7 ± 7.7 | 688 ± 24 | - | 21.24 ± 1.15 | 45.19 ± 4.29 |
| P1604 | 04May2016 | 34.68 | -121.28 | 13.4 ± 0.1 | 4.13 ± 0.25 | 1.00 ± 0.04 | 24.5 ± 6.2 | 895 ± 14 | - | 20.12 ± 1.92 | 75.69 ± 2.53 |
| P1604 | 05May2016 | 34.50 | -121.22 | 13.3 ± 0.1 | 4.41 ± 0.46 | 1.21 ± 0.09 | 36.0 ± 7.7 | 1,706 ± 54 | - | 54.06 ± 2.89 | 144.85 ± 7.05 |
| P1604 | 08May2016 | 34.44 | -120.70 | 13.4 ± 0.5 | 5.52 ± 2.85 | 7.57 ± 1.87 | 88.4 ± 46.1 | 3,642 ± 843 | - | 79.20 ± 25.79 | 49.05 ± 10.27 |
| P1604 | 09May2016 | 34.50 | -120.78 | 13.9 ± 0.3 | 3.00 ± 1.52 | 3.50 ± 0.33 | 114.9 ± 57.2 | 4,397 ± 450 | - | 113.3 ± 16.8 | 69.74 ± 14.29 |
| P1706 | 09June2017 | 35.07 | -121.09 | 12.0 ± 0.1 | 12.09 ± 0.81 | 5.04 ± 1.05 | 27.4 ± 20.2 | 2,056 ± 235 | - | 4.30 ± 0.75 | 36.93 ± 6.46 |
| P1706 | 10June2017 | 35.05 | -121.14 | 12.8 ± 0.0 | 5.29 ± 0.03 | 13.27 ± 0.15 | 199.3 ± 84.8 | 2,961 ± 23 | - | 5.95 ± 0.09 | 44.99 ± 0.39 |
| P1706 | 11June2017 | 34.95 | -121.18 | 13.0 ± 0.0 | 5.66 ± 0.04 | 8.27 ± 0.21 | 268.5 ± 114.0 | 3,027 ± 88 | - | 10.70 ± 0.25 | 43.82 ± 0.60 |
| P1706 | 13June2017 | 34.73 | -121.71 | 12.9 ± 0.0 | 6.83 ± 0.05 | 4.82 ± 0.26 | 111.6 ± 44.4 | 1,004 ± 22 | - | 2.32 ± 0.07 | 22.00 ± 4.41 |
| P1706 | 14June2017 | 34.72 | -121.92 | 12.7 ± 0.0 | 8.32 ± 0.08 | 3.22 ± 0.12 | 240.0 ± 153.7 | 1,044 ± 16 | - | 2.36 ± 0.04 | 14.13 ± 0.50 |
| P1706 | 15June2017 | 34.72 | -122.14 | 12.9 ± 0.0 | 7.78 ± 0.02 | 3.15 ± 0.05 | 274.2 ± 151.8 | 1,038 ± 18 | - | 1.77 ± 0.03 | 13.01 ± 0.46 |
| P1706 | 16June2017 | 34.71 | -122.28 | 13.0 ± 0.0 | 7.67 ± 0.05 | 2.33 ± 0.04 | 133.2 ± 76.1 | 1,009 ± 39 | - | 1.62 ± 0.05 | 13.63 ± 1.65 |
| P1706 | 19June2017 | 34.37 | -123.18 | 13.4 ± 0.1 | 4.74 ± 0.24 | 1.62 ± 0.07 | 73.7 ± 46.2 | 929 ± 13 | - | 9.34 ± 0.34 | 33.89 ± 3.02 |
| P1706 | 20June2017 | 34.20 | -123.11 | 14.2 ± 0.1 | 2.62 ± 0.34 | 0.64 ± 0.02 | 127.5 ± 65.6 | 1,205 ± 37 | - | 6.71 ± 0.62 | 50.23 ± 3.33 |
| P1706 | 21June2017 | 34.09 | -122.95 | 14.9 ± 0.0 | 0.99 ± 0.06 | 0.41 ± 0.02 | 89.3 ± 39.9 | 1,036 ± 28 | - | 4.85 ± 0.25 | 31.19 ± 0.97 |
| P1706 | 24June2017 | 34.40 | -123.07 | 14.4 ± 0.2 | 2.74 ± 0.26 | 0.49 ± 0.12 | 36.0 ± 16.4 | 916 ± 61 | - | 1.30 ± 0.06 | 6.32 ± 1.22 |
| P1706 | 25June2017 | 34.45 | -122.99 | 13.9 ± 0.4 | 4.57 ± 1.44 | 0.31 ± 0.03 | 299.0 ± 152.5 | 912 ± 66 | - | 1.50 ± 0.32 | 5.28 ± 0.43 |

**Table S3.** Measured rates of population growth (μ, $d^{-1}$) and grazing mortality (m, $d^{-1}$) for heterotrophic bacteria (HBAC), *Prochlorococcus* (PRO), *Synechococcus* (SYN) and photosynthetic picoeukaryotes (PEUK) in the California Current Ecosystem. Data are averaged for experiments in the upper euphotic zone. Uncertainties are standard errors of mean (SEM) values.

| Cruise | Date | Lat (°N) | Long (°W) | HBAC μ ($d^{-1}$) | HBAC m ($d^{-1}$) | PRO μ ($d^{-1}$) | PRO m ($d^{-1}$) | SYN μ ($d^{-1}$) | SYN m ($d^{-1}$) | PEUK μ ($d^{-1}$) | PEUK m ($d^{-1}$) |
|---|---|---|---|---|---|---|---|---|---|---|---|
| P0605 | 11May2006 | 34.33 | -120.80 | 0.513 ± 0.027 | 0.316 ± 0.032 | ND | ND | 0.318 ± 0.067 | 0.113 ± 0.085 | 0.569 ± 0.101 | 0.112 ± 0.064 |
| P0605 | 12May2006 | 34.29 | -120.84 | 0.778 ± 0.026 | 0.467 ± 0.054 | ND | ND | 0.552 ± 0.040 | 0.171 ± 0.086 | 0.791 ± 0.111 | 0.326 ± 0.073 |
| P0605 | 13May2006 | 34.29 | -120.98 | 0.772 ± 0.023 | 0.487 ± 0.012 | ND | ND | 0.492 ± 0.048 | 0.220 ± 0.071 | 0.743 ± 0.078 | 0.334 ± 0.035 |
| P0605 | 14May2006 | 34.26 | -121.06 | 0.770 ± 0.018 | 0.485 ± 0.043 | ND | ND | 0.458 ± 0.053 | 0.230 ± 0.118 | 0.590 ± 0.262 | 0.381 ± 0.067 |
| P0605 | 17May2006 | 33.50 | -122.11 | 0.207 ± 0.024 | 0.141 ± 0.031 | 0.266 ± 0.127 | 0.170 ± 0.155 | 2.139 ± 0.651 | 2.326 ± 0.874 | 0.620 ± 0.031 | 0.653 ± 0.037 |
| P0605 | 19May2006 | 33.62 | -122.13 | 0.138 ± 0.029 | 0.073 ± 0.036 | (0.184) ± 0.207 | 0.179 ± 0.050 | 0.552 ± 0.129 | 0.447 ± 0.126 | 0.328 ± 0.043 | 0.317 ± 0.099 |
| P0605 | 20May2006 | 33.49 | -122.09 | 0.145 ± 0.017 | 0.117 ± 0.060 | (0.267) ± 0.103 | 0.185 ± 0.104 | 0.901 ± -.311 | 0.656 ± 0.274 | 0.224 ± 0.129 | 0.203 ± 0.083 |
| P0605 | 22May2006 | 34.63 | -120.77 | 1.008 ± 0.127 | 1.077 ± 0.090 | ND | ND | 0.469 ± 0.009 | 0.452 ± 0.039 | 0.454 ± 0.134 | 0.427 ± 0.083 |
| P0605 | 23May2006 | 34.75 | -120.77 | 0.767 ± 0.174 | 0.732 ± 0.204 | ND | ND | 0.555 ± 0.088 | 0.369 ± 0.121 | 0.361 ± 0.177 | 0.258 ± 0.109 |
| P0605 | 24May2006 | 34.86 | -120.80 | 1.204 ± 0.078 | 1.214 ± 0.081 | ND | ND | 0.436 ± 0.066 | 0.372 ± 0.079 | 0.605 ± 0.179 | 0.204 ± 0.118 |
| P0605 | 26May2006 | 34.05 | -121.30 | 0.326 ± 0.010 | 0.255 ± 0.049 | ND | ND | 0.323 ± 0.038 | 0.192 ± 0.022 | 0.507 ± 0.029 | 0.239 ± 0.008 |
| P0605 | 27May2006 | 34.02 | -121.43 | 0.206 ± 0.022 | 0.166 ± 0.025 | ND | ND | 0.450 ± 0.037 | 0.158 ± 0.057 | 0.690 ± 0.037 | 0.198 ± 0.020 |
| P0605 | 28May2006 | 33.96 | -121.57 | 0.471 ± 0.032 | 0.316 ± 0.061 | ND | ND | 0.286 ± 0.020 | 0.216 ± 0.036 | 0.561 ± 0.039 | 0.325 ± 0.033 |
| P0605 | 30May2006 | 33.87 | -121.72 | 0.569 ± 0.040 | 0.215 ± 0.069 | ND | ND | 0.379 ± 0.014 | 0.238 ± 0.057 | 0.670 ± 0.038 | 0.252 ± 0.029 |
| P0605 | 01June2006 | 32.85 | -124.00 | 0.100 ± 0.071 | 0.073 ± 0.093 | 0.828 ± 0.249 | 0.499 ± 0.315 | 0.436 ± 0.057 | 0.608 ± 0.096 | 0.390 ± 0.186 | 0.164 ± 0.086 |
| P0605 | 02June2006 | 32.78 | -124.09 | 0.111 ± 0.921 | 0.071 ± 0.019 | 0.515 ± 0.089 | 0.236 ± 0.060 | 0.448 ± 0.060 | 0.361 ± 0.099 | 0.293 ± 0.213 | 0.342 ± 0.250 |
| P0605 | 03June2006 | 32.67 | -124.19 | 0.114 ± 0.020 | 0.080 ± 0.042 | 0.383 ± 0.065 | 0.273 ± 0.047 | 0.599 ± 0.096 | 0.731 ± 0.195 | 0.000 ± 0.070 | (0.255) ± 0.097 |
| P0605 | 04June2006 | 32.49 | -124.25 | 0.272 ± 0.041 | 0.118 ± 0.039 | (0.057) ± 0.091 | 0.279 ± 0.046 | 0.722 ± 0.111 | 0.596 ± 0.087 | 0.522 ± 0.164 | 0.224 ± 0.099 |
| P0704 | 05Apr2007 | 34.28 | -120.91 | 0.458 ± 0.074 | 0.283 ± 0.056 | ND | ND | 0.611 ± 0.065 | 0.436 ± 0.099 | 1.048 ± 0.066 | 0.455 ± 0.069 |
| P0704 | 06Apr2007 | 34.33 | -120.95 | 0.230 ± 0.056 | 0.217 ± 0.037 | ND | ND | 0.513 ± 0.056 | 0.326 ± 0.098 | 0.676 ± 0.121 | 0.513 ± 0.033 |
| P0704 | 07Apr2007 | 34.36 | -121.05 | 0.352 ± 0.069 | 0.325 ± 0.095 | ND | ND | 0.661 ± 0.072 | 0.596 ± 0.119 | 0.991 ± 0.060 | 0.737 ± 0.119 |

| | | | | | | | | | | | |
|---|---|---|---|---|---|---|---|---|---|---|---|
| P0704 | 10Apr2007 | 33.55 | -123.16 | 0.042 ± 0.046 | 0.131 ± 0.010 | 0.488 ± 0.069 | 0.235 ± 0.022 | 0.202 ± 0.129 | 0.208 ± 0.074 | 0.156 ± 0.022 | 0.121 ± 0.045 |
| P0704 | 11Apr2007 | 33.41 | -123.28 | (0.013) ± 0.021 | 0.097 ± 0.023 | 0.956 ± 0.019 | 0.743 ± 0.040 | 0.305 ± 0.079 | 0.308 ± 0.051 | 0.136 ± 0.063 | 0.252 ± 0.053 |
| P0704 | 12Apr2007 | 33.33 | -123.45 | 0.025 ± 0.016 | 0.166 ± 0.019 | 0.559 ± 0.095 | 0.491 ± 0.104 | 0.183 ± 0.104 | 0.278 ± 0.100 | 0.223 ± 0.081 | 0.327 ± 0.072 |
| P0704 | 15Apr2007 | 34.22 | -120.62 | 0.408 ± 0.018 | 0.223 ± 0.006 | ND | ND | 0.082 ± 0.055 | 0.089 ± 0.039 | 0.196 ± 0.128 | 0.325 ± 0.120 |
| P0704 | 16Apr2007 | 34.23 | -121.19 | 0.412 ± 0.022 | 0.255 ± 0.029 | ND | ND | 0.566 ± 0.101 | 0.383 ± 0.097 | (0.523) ± 0.102 | 0.393 ± 0.099 |
| P0704 | 17Apr2007 | 33.92 | -121.16 | 0.140 ± 0.056 | 0.076 ± 0.016 | ND | ND | 0.172 ± 0.087 | (0.019) ± 0.023 | 0.609 ± 0.075 | 0.242 ± 0.110 |
| P0704 | 18Apr2007 | 33.82 | -121.08 | 0.321 ± 0.035 | 0.358 ± 0.061 | ND | ND | 0.516 ± 0.034 | 0.288 ± 0.094 | 0.783 ± 0.127 | 0.466 ± 0.133 |
| P0704 | 19Apr2007 | 33.74 | -121.00 | 0.114 ± 0.032 | 0.140 ± 0.046 | ND | ND | 0.740 ± 0.087 | 0.448 ± 0.061 | 0.760 ± 0.114 | 0.246 ± 0.135 |
| P0810 | 04Oct2008 | 34.13 | -120.98 | 0.428 ± 0.047 | 0.642 ± 0.115 | 0.257 ± 0.072 | 0.184 ± 0.080 | 0.415 ± 0.082 | 0.430 ± 0.132 | 0.356 ± 0.131 | 0.187 ± 0.052 |
| P0810 | 05Oct2008 | 34.11 | -120.84 | 0.354 ± 0.157 | 0.330 ± 0.109 | 0.253 ± 0.081 | 0.268 ± 0.094 | 0.444 ± 0.097 | 0.276 ± 0.135 | 0.667 ± 0.410 | 0.266 ± 0.135 |
| P0810 | 06Oct2008 | 33.97 | -120.77 | 0.727 ± 0.081 | 0.499 ± 0.109 | 0.691 ± 0.152 | 0.552 ± 0.140 | 0.213 ± 0.038 | 0.279 ± 0.060 | 0.136 ± 0.044 | 0.159 ± 0.088 |
| P0810 | 07Oct2008 | 33.78 | -120.73 | 0.637 ± 0.214 | 0.530 ± 0.170 | 0.579 ± 0.197 | 0.551 ± 0.178 | 0.270 ± 0.068 | 0.511 ± 0.088 | 0.307 ± 0.100 | 0.461 ± 0.132 |
| P0810 | 09Oct2008 | 32.89 | -123.69 | 0.588 ± 0.155 | 0.403 ± 0.225 | 0.624 ± 0.271 | 0.262 ± 0.251 | 0.426 ± 0.244 | 0.331 ± 0.256 | 0.308 ± 0.105 | 0.264 ± 0.140 |
| P0810 | 10Oct2008 | 32.80 | -123.66 | 0.356 ± 0.170 | 0.018 ± 0.089 | 0.358 ± 0.062 | 0.314 ± 0.041 | 0.194 ± 0.127 | 0.153 ± 0.111 | 0.116 ± 0.080 | 0.067 ± 0.098 |
| P0810 | 12Oct2008 | 32.45 | -123.73 | 0.304 ± 0.167 | 0.560 ± 0.143 | 0.528 ± 0.116 | 0.398 ± 0.052 | 0.092 ± 0.056 | 0.071 ± 0.029 | 0.092 ± 0.075 | 0.123 ± 0.076 |
| P0810 | 13Oct2008 | 32.29 | -123.84 | 0.286 ± 0.080 | 0.156 ± 0.096 | 0.440 ± 0.065 | 0.374 ± 0.104 | 0.228 ± 0.096 | 0.368 ± 0.136 | 0.290 ± 0.084 | 0.305 ± 0.071 |
| P0810 | 16Oct2008 | 34.02 | -121.76 | 0.425 ± 0.014 | 0.170 ± 0.036 | 0.270 ± 0.073 | 0.205 ± 0.060 | 0.404 ± 0.119 | 0.177 ± 0.065 | 0.343 ± 0.030 | 0.192 ± 0.066 |
| P0810 | 17Oct2008 | 34.20 | -121.58 | 0.336 ± 0.022 | 0.271 ± 0.020 | 0.423 ± 0.068 | 0.272 ± 0.085 | 0.206 ± 0.036 | 0.121 ± 0.035 | 0.228 ± 0.033 | 0.255 ± 0.066 |
| P0810 | 19Oct2008 | 33.61 | -121.15 | 0.028 ± 0.107 | 0.171 ± 0.088 | ND | ND | 0.630 ± 0.147 | 0.421 ± 0.066 | (0.034) ± 0.094 | 0.123 ± 0.067 |
| P0810 | 20Oct2008 | 33.61 | -121.16 | 0.448 ± 0.044 | 0.283 ± 0.068 | ND | ND | 0.442 ± 0.199 | 0.425 ± 0.206 | 0.111 ± 0.057 | 0.211 ± 0.076 |
| P0810 | 22Oct2008 | 32.92 | -120.90 | 0.223 ± 0.027 | 0.254 ± 0.025 | ND | ND | 0.503 ± 0.085 | 0.291 ± 0.048 | 0.652 ± 0.144 | 0.428 ± 0.057 |
| P0810 | 23Oct2008 | 32.82 | -120.84 | 0.625 ± 0.049 | 0.462 ± 0.057 | ND | ND | 0.464 ± 0.057 | 0.403 ± 0.065 | 0.464 ± 0.105 | 0.374 ± 0.094 |
| P0810 | 26Oct2008 | 32.60 | -120.56 | 0.029 ± 0.030 | 0.034 ± 0.042 | 0.473 ± 0.034 | 0.255 ± 0.041 | 0.291 ± 0.100 | 0.236 ± 0.105 | 0.187 ± 0.065 | 0.175 ± 0.027 |
| P0810 | 27Oct2008 | 32.57 | -120.33 | 0.150 ± 0.038 | 0.124 ± 0.048 | 0.239 ± 0.076 | 0.180 ± 0.102 | 0.250 ± 0.091 | 0.292 ± 0.146 | 0.155 ± 0.007 | 0.219 ± 0.031 |
| P1106 | 24June2011 | 34.03 | -121.70 | 0.511 ± 0.049 | 0.438 ± 0.218 | 0.238 ± 0.202 | 0.191 ± 0.235 | 0.298 ± 0.045 | 0.246 ± 0.082 | 0.603 ± 0.106 | 0.655 ± 0.204 |
| P1106 | 25June2011 | 33.91 | -121.46 | 0.466 ± 0.072 | 0.485 ± 0.054 | 0.618 ± 0.124 | 0.619 ± 0.157 | 0.280 ± 0.019 | 0.224 ± 0.044 | 0.560 ± 0.031 | 0.226 ± 0.012 |
| P1106 | 27June2011 | 33.58 | -121.77 | 0.328 ± 0.104 | 0.127 ± 0.152 | 0.916 ± 0.121 | 0.415 ± 0.264 | 0.082 ± 0.151 | 0.262 ± 0.144 | 0.114 ± 0.326 | 0.479 ± 0.316 |
| P1106 | 28June2011 | 33.42 | -121.62 | 0.343 ± 0.152 | 0.234 ± 0.102 | 0.284 ± 0.060 | 0.341 ± 0.062 | 0.294 ± 0.072 | 0.572 ± 0.183 | 0.500 ± 0.040 | 0.559 ± 0.043 |
| P1106 | 30June2011 | 34.11 | -121.65 | 0.748 ± 0.096 | 0.590 ± 0.094 | 0.835 ± 0.116 | 0.403 ± 0.100 | 0.337 ± 0.133 | 0.190 ± 0.121 | 0.587 ± 0.089 | 0.382 ± 0.073 |

| | | | | | | | | | | | |
|---|---|---|---|---|---|---|---|---|---|---|---|
| P1106 | 01July2011 | 34.06 | -121.47 | 0.164 ± 0.051 | 0.169 ± 0.049 | 0.670 ± 0.089 | 0.427 ± 0.088 | 0.383 ± 0.087 | 0.234 ± 0.081 | 0.644 ± 0.063 | 0.389 ± 0.054 |
| P1106 | 07July2011 | 33.53 | -121.09 | 0.038 ± 0.129 | 0.409 ± 0.087 | 0.670 ± 0.133 | 0.699 ± 0.130 | 0.153 ± 0.113 | 0.607 ± 0.072 | (0.198) ± 0.128 | 0.470 ± 0.118 |
| P1106 | 08July2011 | 33.39 | -121.16 | 1.208 ± 0.116 | 0.561 ± 0.099 | ND | ND | 0.386 ± 0.144 | 0.587 ± 0.110 | 0.475 ± 0.143 | 0.646 ± 0.100 |
| P1106 | 10July2011 | 33.42 | -121.39 | 0.108 ± 0.124 | 0.075 ± 0.100 | 1.351 ± 0.143 | 1.445 ± 0.118 | 0.241 ± 0.241 | 0.476 ± 0.303 | 0.430 ± 0.198 | 0.522 ± 0.158 |
| P1106 | 11July2011 | 33.08 | -121.41 | 0.232 ± 0.044 | 0.276 ± 0.028 | 0.322 ± 0.150 | 0.167 ± 0.160 | 0.196 ± 0.084 | 0.511 ± 0.274 | (0.033) ± 0.113 | 0.142 ± 0.125 |
| P1106 | 13July2011 | 33.52 | -121.11 | 0.480 ± 0.049 | 0.321 ± 0.036 | 0.521 ± 0.132 | 0.360 ± 0.137 | 0.148 ± 0.104 | 0.274 ± 0.141 | 0.459 ± 0.167 | 0.300 ± 0.154 |
| P1106 | 14July2011 | 33.07 | -121.26 | 0.365 ± 0.124 | 0.467 ± 0.133 | 0.738 ± 0.066 | 0.620 ± 0.056 | 0.217 ± 0.109 | 0.252 ± 0.082 | 0.483 ± 0.101 | 0.398 ± 0.062 |
| P1208 | 06Aug2012 | 34.56 | -122.77 | 0.542 ± 0.018 | 0.303 ± 0.035 | 0.281 ± 0.061 | 0.184 ± 0.081 | 0.289 ± 0.102 | 0.107 ± 0.031 | 0.241 ± 0.154 | 0.263 ± 0.066 |
| P1208 | 07Aug2012 | 34.21 | -122.90 | 0.474 ± 0.054 | 0.272 ± 0.043 | 0.140 ± 0.165 | 0.042 ± 0.182 | 0.233 ± 0.102 | 0.017 ± 0.021 | 0.363 ± 0.166 | 0.266 ± 0.109 |
| P1208 | 08Aug2012 | 33.85 | -122.96 | 0.425 ± 0.004 | 0.311 ± 0.012 | 0.874 ± 0.350 | 0.510 ± 0.046 | 0.168 ± 0.042 | 0.175 ± 0.007 | 0.245 ± 0.163 | 0.408 ± 0.061 |
| P1208 | 10Aug2012 | 34.57 | -123.04 | 0.164 ± 0.034 | 0.137 ± 0.037 | 0.296 ± 0.063 | 0.227 ± 0.057 | 0.112 ± 0.067 | 0.136 ± 0.051 | 0.332 ± 0.004 | 0.302 ± 0.015 |
| P1208 | 11Aug2012 | 34.09 | -123.26 | 0.193 ± 0.073 | 0.283 ± 0.160 | 0.133 ± 0.048 | 0.120 ± 0.048 | 0.169 ± 0.070 | 0.210 ± 0.077 | 0.263 ± 0.130 | 0.293 ± 0.124 |
| P1208 | 13Aug2012 | 34.54 | -122.50 | 0.252 ± 0.036 | 0.313 ± 0.038 | 0.897 ± 0.116 | 0.613 ± 0.117 | 0.273 ± 0.066 | 0.331 ± 0.031 | 0.393 ± 0.105 | 0.161 ± 0.078 |
| P1208 | 14Aug2012 | 34.50 | -122.48 | 0.195 ± 0.203 | 0.203 ± 0.047 | 0.309 ± 0.033 | 0.133 ± 0.042 | 0.240 ± 0.090 | 0.262 ± 0.076 | 0.309 ± 0.077 | 0.233 ± 0.010 |
| P1208 | 16Aug2012 | 34.54 | -123.27 | 0.242 ± 0.048 | 0.190 ± 0.029 | 0.266 ± 0.081 | 0.271 ± 0.085 | 0.206 ± 0.021 | 0.232 ± 0.081 | 0.357 ± 0.194 | 0.433 ± 0.162 |
| P1208 | 17Aug2012 | 34.31 | -123.46 | 0.380 ± 0.076 | 0.109 ± 0.056 | 0.175 ± 0.161 | 0.018 ± 0.180 | 0.322 ± 0.081 | 0.150 ± 0.108 | (0.107) ± 0.080 | 0.097 ± 0.061 |
| P1208 | 19Aug2012 | 34.08 | -123.32 | 0.420 ± 0.039 | 0.321 ± 0.045 | 0.216 ± 0.052 | 0.156 ± 0.043 | 0.273 ± 0.028 | 0.194 ± 0.043 | 0.339 ± 0.092 | 0.403 ± 0.089 |
| P1408 | 11Aug2014 | 34.51 | -120.77 | 0.671 ± 0.093 | 0.655 ± 0.109 | 0.482 ± 0.286 | 0.774 ± 0.217 | 0.668 ± 0.114 | 0.363 ± 0.102 | 0.522 ± 0.117 | 0.522 ± 0.159 |
| P1408 | 12Aug2014 | 34.72 | -121.00 | 0.360 ± 0.042 | 0.410 ± 0.054 | 2.135 ± 0.375 | 1.213 ± 0.197 | (0.051) ± 0.239 | (0.144) ± 0.253 | 0.411 ± 0.109 | 0.547 ± 0.117 |
| P1408 | 13Aug2014 | 34.81 | -121.22 | 0.838 ± 0.070 | 0.645 ± 0.040 | 0.789 ± 0.391 | (0.019) ± 0.504 | 0.749 ± 0.097 | 0.722 ± 0.150 | 0.670 ± 0.066 | 0.823 ± 0.142 |
| P1408 | 17Aug2014 | 34.27 | -120.82 | 0.599 ± 0.080 | 0.785 ± 0.131 | 0.571 ± 0.211 | 0.017 ± 0.044 | 0.430 ± 0.095 | 0.516 ± 0.183 | 0.145 ± 0.097 | 0.277 ± 0.081 |
| P1408 | 18Aug2014 | 34.17 | -120.87 | 0.379 ± 0.084 | 0.415 ± 0.039 | 0.278 ± 0.130 | 0.139 ± 0.135 | 0.567 ± 0.156 | 0.597 ± 0.101 | 0.426 ± 0.150 | 0.434 ± 0.143 |
| P1408 | 19Aug2014 | 34.12 | -120.92 | 0.452 ± 0.013 | 0.398 ± 0.044 | 0.373 ± 0.127 | 0.470 ± 0.083 | 0.534 ± 0.131 | 0.605 ± 0.124 | 0.448 ± 0.093 | 0.490 ± 0.084 |
| P1408 | 22Aug2014 | 34.39 | -121.39 | 0.261 ± 0.046 | 0.112 ± 0.040 | 0.853 ± 0.118 | 0.619 ± 0.160 | 0.284 ± 0.030 | 0.263 ± 0.054 | 0.291 ± 0.088 | 0.311 ± 0.070 |
| P1408 | 23Aug2014 | 34.41 | -121.28 | 0.301 ± 0.132 | 0.348 ± 0.102 | 0.837 ± 0.150 | 0.916 ± 0.082 | 0.205 ± 0.082 | 0.288 ± 0.089 | 0.637 ± 0.167 | 0.694 ± 0.137 |
| P1408 | 24Aug2014 | 34.43 | -121.15 | 0.258 ± 0.021 | 0.439 ± 0.027 | 0.836 ± 0.054 | 0.639 ± 0.053 | 0.445 ± 0.054 | 1.046 ± 0.282 | 0.472 ± 0.146 | 0.570 ± 0.158 |
| P1408 | 26Aug2014 | 33.52 | -122.56 | 0.246 ± 0.040 | 0.272 ± 0.076 | 0.110 ± 0.074 | 0.029 ± 0.080 | 0.125 ± 0.231 | 1.130 ± 0.539 | 0.530 ± 0.334 | 0.631 ± 0.367 |
| P1408 | 27Aug2014 | 33.54 | -122.51 | 0.214 ± 0.025 | 0.246 ± 0.043 | 0.038 ± 0.075 | 0.100 ± 0.080 | 1.405 ± 0.150 | 1.933 ± 0.212 | 1.053 ± 0.358 | 0.937 ± 0.217 |
| P1408 | 28Aug2014 | 33.60 | -122.44 | 0.230 ± 0.052 | 0.289 ± 0.088 | 0.283 ± 0.026 | 0.243 ± 0.048 | 1.351 ± 0.235 | 1.142 ± 0.458 | 0.350 ± 0.140 | 0.579 ± 0.156 |

| | | | | | | | | | | | |
|---|---|---|---|---|---|---|---|---|---|---|---|
| P1408 | 30Aug2014 | 32.88 | -123.90 | 0.336 ± 0.059 | 0.324 ± 0.083 | (0.089) ± 0.129 | (0.125) ± 0.134 | 0.744 ± 0.189 | 0.993 ± 0.417 | 2.589 ± 0.416 | 2.489 ± 0.409 |
| P1408 | 31Aug2014 | 32.84 | -123.87 | 0.323 ± 0.055 | 0.449 ± 0.133 | 0.108 ± 0.084 | 0.109 ± 0.076 | 0.520 ± 0.091 | 1.034 ± 0.156 | 0.366 ± 1.029 | 1.183 ± 0.642 |
| P1408 | 01Sept2014 | 32.81 | -123.87 | 0.297 ± 0.016 | 0.513 ± 0.084 | 1.007 ± 0.029 | 1.223 ± 0.044 | 0.428 ± 0.281 | 0.845 ± 0.239 | 0.415 ± 0.199 | 0.665 ± 0.309 |
| P1604 | 22Apr2016 | 33.09 | -122.94 | 0.108 ± 0.061 | 0.096 ± 0.068 | 0.176 ± 0.089 | 0.102 ± 0.071 | 0.460 ± 0.140 | 0.351 ± 0.104 | 0.914 ± 0.349 | 0.739 ± 0.306 |
| P1604 | 29Apr2016 | 33.59 | -122.21 | 0.224 ± 0.086 | 0.231 ± 0.060 | 0.505 ± 0.084 | 0.605 ± 0.134 | 0.424 ± 0.102 | 0.305 ± 0..098 | 0.757 ± 0.305 | 0.660 ± 0.328 |
| P1604 | 30Apr2016 | 33.52 | -122.06 | 0.262 ± 0.038 | 0.226 ± 0.052 | 0.088 ± 0.019 | 0.055 ± 0.017 | 0.577 ± 0.104 | 0.408 ± 0.048 | 0.199 ± 0.179 | 0.048 ± 0.211 |
| P1604 | 01May2016 | 33.47 | -121.97 | 0.187 ± 0.050 | 0.197 ± 0.056 | 0.261 ± 0.055 | 0.230 ± 0.045 | 0.371 ± 0.082 | 0.308 ± 0.133 | 0.286 ± 0.138 | 0.320 ± 0.144 |
| P1604 | 03May2016 | 34.80 | -121.28 | 0.410 ± 0.043 | 0.315 ± 0.036 | ND | ND | 0.469 ± 0.134 | 0.510 ± 0.038 | 0.734 ± 0.126 | 0.623 ± 0.060 |
| P1604 | 04May2016 | 34.68 | -121.28 | 0.380 ± 0.037 | 0.284 ± 0.014 | ND | ND | 0.460 ± 0.128 | 0.233 ± 0.064 | 0.615 ± 0.169 | 0.431 ± 0.076 |
| P1604 | 05May2016 | 34.50 | -121.22 | 0.323 ± 0.035 | 0.099 ± 0.039 | ND | ND | 0.552 ± 0.071 | 0.092 ± 0.041 | 0.667 ± 0.104 | 0.133 ± 0.047 |
| P1604 | 08May2016 | 34.44 | -120.70 | 0.428 ± 0.084 | 0.295 ± 0.041 | ND | ND | 0.270 ± 0.121 | 0.085 ± 0.019 | 0.472 ± 0.069 | 0.272 ± 0.200 |
| P1604 | 09May2016 | 34.50 | -120.78 | 0.386 ± 0.053 | 0.450 ± 0.045 | ND | ND | 0.300 ± 0.145 | 0.109 ± 0.045 | 0.360 ± 0.266 | 0.354 ± 0.161 |
| P1706 | 09June2017 | 35.07 | -121.09 | 0.448 ± 0.057 | 0.308 ± 0.019 | ND | ND | 0.234 ± 0.184 | 0.087 ± 0.124 | 0.265 ± 0.188 | 0.233 ± 0.038 |
| P1706 | 10June2017 | 35.05 | -121.14 | 0.675 ± 0.117 | 0.388 ± 0.161 | ND | ND | 0.165 ± 0.045 | (0.064) ± 0.066 | 0.354 ± 0.191 | 0.411 ± 0.085 |
| P1706 | 11June2017 | 34.95 | -121.18 | 0.798 ± 0.079 | 0.477 ± 0.072 | ND | ND | 0.266 ± 0.063 | 0.091 ± 0.067 | 0.745 ± 0.057 | 0.739 ± 0.109 |
| P1706 | 13June2017 | 34.73 | -121.71 | 1.152 ± 0.070 | 1.031 ± 0.054 | ND | ND | 0.391 ± 0.134 | 0.270 ± 0.106 | 0.359 ± 0.319 | 0.462 ± 0.152 |
| P1706 | 14June2017 | 34.72 | -121.92 | 1.057 ± 0.058 | 0.810 ± 0.028 | ND | ND | 0.197 ± 0.055 | 0.013 ± 0.102 | 0.158 ± 0.208 | 0.048 ± 0.145 |
| P1706 | 15June2017 | 34.72 | -122.14 | 1.109 ± 0.051 | 0.885 ± 0.052 | ND | ND | 0.370 ± 0.158 | 0.287 ± 0.079 | 0.296 ± 0.226 | 0.167 ± 0.157 |
| P1706 | 16June2017 | 34.71 | -122.28 | 1.039 ± 0.067 | 0.758 ± 0.087 | ND | ND | 0.368 ± 0.053 | 0.100 ± 0.097 | 0.030 ± 0.103 | 0.206 ± 0.081 |
| P1706 | 19June2017 | 34.37 | -123.18 | 0.917 ± 0.098 | 0.620 ± 0.028 | ND | ND | 0.489 ± 0.074 | 0.434 ± 0.024 | 0.584 ± 0.108 | 0.547 ± 0.035 |
| P1706 | 20June2017 | 34.20 | -123.11 | 0.495 ± 0.061 | 0.311 ± 0.020 | ND | ND | 0.329 ± 0.096 | 0.113 ± 0.095 | 0.209 ± 0.140 | 0.117 ± 0.067 |
| P1706 | 21June2017 | 34.09 | -122.95 | 0.301 ± 0.054 | 0.127 ± 0.053 | ND | ND | 0.301 ± 0.026 | 0.079 ± 0.028 | 0.304 ± 0.130 | (0.072) ± 0.040 |
| P1706 | 24June2017 | 34.40 | -123.07 | 0.554 ± 0.054 | 0.201 ± 0.016 | ND | ND | 0.393 ± 0.102 | (0.026) ± 0.046 | 0.521 ± 0.291 | 0.026 ± 0.120 |
| P1706 | 25June2017 | 34.45 | -122.99 | 0.467 ± 0.042 | 0.294 ± 0.016 | ND | ND | 0.682 ± 0.104 | 0.244 ± 0.222 | 0.800 ± 0.116 | 0.272 ± 0.276 |
